# Supplementary material for: Genome-wide association study in 79,366 European-ancestry individuals informs the genetic architecture of 25-hydroxyvitamin D levels
Source: Nat Commun. 2018 Jan 17;9:260. doi: 10.1038/s41467-017-02662-2 (PMC5772647; doi:10.1038/s41467-017-02662-2)
Supplement: Supplementary file 1 — Supplementary Information [file 41467_2017_2662_MOESM1_ESM.pdf]

Supplementary Note 1

Supplemental Methods

Overview of consortium

The SUNLIGHT consortium (Study of Underlying Genetic Determinants of Vitamin D and Highly Related Traits) was formed in 2008. It represents a collaboration of more than 30 studies from the United Kingdom, United States, Canada, Netherlands, Germany, Sweden, Italy, and Finland. Fifteen studies had joined SUNLIGHT consortium as of 2010, ten of which, were used in the current analysis, and were described in our earlier GWAS meta-analysis.[1] Additional cohorts joined the consortium since that time, resulting in a significant increase in the sample size. We included 21 of those additional cohorts in our current overall meta-GWAS analysis. Four of the participating cohorts (Atherosclerosis Risk in Communities Study, Framingham Heart Study, Cardiovascular Health Study, and the Rotterdam Study) are also members of the CHARGE consortium (Cohorts for Heart and Aging Research in Genetic Epidemiology).[2]

Individual cohort descriptions

The characteristic of the 10 individual cohorts (the **1958 British Birth Cohort Study (1958BC)**, the **Cardiovascular Health Study (CHS)**, the **Framingham Heart Study (FHS)**, the **Gothenburg Osteoporosis and Obesity Determinants Study (GOOD)**, the **Health, Aging and Body Composition Study (Health ABC)**, the **Indiana Women Cohort**, the **Northern Finland Birth Cohort 1966 (NFBC1966)**, the **Old order Amish Study (OOA)**, the **Rotterdam Study (RS)**, and the **TwinsUK registry**) were previously described in detail.[1] The 21 additional cohorts are briefly described below, as well as the 2 cohorts that were used only in the gene-by-dietary vitamin D intake interaction analysis (but not in the overall meta-GWAS analysis) as follows.

The **Alpha-Tocopherol, Beta-Carotene Cancer Prevention Trial** (ATBC) was a randomized, placebo-controlled, double-blind cancer prevention trial conducted by the US National Cancer Institute and the National Institute for Health and Welfare of Finland from 1985 to 1993. A total of 29,133 eligible male smokers participated in the study and follow-up for cancer endpoints is conducted with linkage to the Finnish Cancer Registry and cancer reviews. For the current study, follow-up is through 2012. Participants completed a general risk factor, smoking and medical history questionnaire along with a food frequency questionnaire at baseline. Fasting serum samples were collected at the bassline visit, and a whole blood sample was obtained close to the end of the trial.[3] Genome-wide genotyping was performed using the Illumina 550K.[4] The study was approved by the Institutional Review Boards of the National Cancer Institute and the National Public Health Institute of Finland.

The **Atherosclerosis Risk in Communities** (ARIC) Study is a prospective epidemiologic study conducted in four US communities (Washington County, MD; Forsyth County, NC; Jackson, MS; and Minneapolis, MN) to investigate the etiology of atherosclerosis and its clinical consequences and variation in cardiovascular risk factors, medical care, and disease by race, gender, location and date. ARIC began in 1987, each of the four ARIC field centers randomly recruited a cohort sample of approximately 4,000 individuals aged 45-64 from a defined population in their community, to receive extensive examinations. A total of 15,792 participants were included in the baseline; several follow-up visits have taken place.[5] Blood samples used for vitamin D measurement were collected in 1990-1992. Genotyping of SNPs was performed using the Affymetrix 6.0 microarray. The study protocols were reviewed and approved by each site's Institutional Review Board at The Johns Hopkins University, Wake Forest University, University of Mississippi Medical Center, and University of Minnesota.

The **AtheroGene** Registry is a cardiovascular registry initiated in 1996 in Germany, enrolling patients who underwent coronary angiography at the Johannes Gutenberg University Hospital in Mainz. Blood was drawn from all study participants under standardized conditions before coronary angiography was performed.[6] Genotyping was performed using Illumina array. The study protocols were reviewed and approved by the local Ethics Committees at the Johannes Gutenberg-University Mainz and the Federal Armed Forces Central Hospital, Koblenz.

The **B-Vitamins for the Prevention of Osteoporotic Fractures** (B-PROOF) Study is a randomized, placebo-controlled, double-blind parallel intervention study, carried out by a consortium in the Netherlands to understand the association between vitamin B, folate status, and fracture risk. Recruitment took place from 2008 until 2011, via the registries of municipalities in the area of the research centers by inviting all inhabitants aged > 65 years by mail. A total of 2,919 individuals participated the trial. Measurements covering bone health, nutritional intake and status, cognitive function, depression, genetics and quality of life, were performed at the baseline and after two years.[7] Genotyping was performed using the IlluminaOmniExpression array. The study protocols were reviewed and approved by the Medical Ethics committees of Wageningen University and the Erasmus MC and VU University Medical Center.

The **Epidemiology of Diabetes Interventions and Complications** (EDIC) Study is a multicenter, longitudinal, observational study designed to use the well-characterized Diabetes Control and Complications Trial patients to determine the long-term effects of prior separation of glycemic levels on micro- and macrovascular outcomes of type 1 diabetes. It was initiated in 1994 in the US, and recruited 1,375 participants at the baseline. Each subject has a standardized annual history and physical examination. DNA has been obtained from peripheral blood leukocytes in all subjects and stored as a long-term resource for further analysis.[8] Genotyping was performed with the Human 1M BeadChip.[9] The study protocols were reviewed and approved by Institutional Review Boards at all participating centers.

The Health 2000 Survey was a comprehensive combination of health interview and health examination survey carried out in 2000-2001. It aims to obtain information on the most important public health problems in working-aged and aged population, their causes and treatments, as well as on the population's functional and working capacity. The study collected nationally representative sample of 8,028 individuals aged > 30 living in the mainland Finland, and an additional sample of ~3,100 individuals from the Mini-Finland Health Examination Survey. The **Case-Control Study for Metabolic Syndrome** (GENMETS) sub-cohort with 1,641 individuals was part of the Health 2000 survey, selected as case-control study for metabolic syndrome for genome-wide genotyping, performed at the Illumina 610K platform.[10] The study protocols were reviewed and approved by the Coordinating Ethics Committee at the Hospital District of Helsinki and Uusimaa.

The **Helsinki Birth Cohort Study** (HBCS, 1934-44) is a unique, longitudinal epidemiological cohort including individuals born in Finland, and with extensive data throughout the life span including prenatal, early childhood and later life. Over 2000 randomly selected voluntary participants have been followed up clinically for over one decade with more detailed and extensive phenotypic data, dietary information, psychological factors, and genetic data (genotyped with Illumina 670K) available.[11] The study protocols were reviewed and approved by the Coordinating Ethics Committee at the Hospital District of Helsinki and Uusimaa.

The **Health Professionals Fellow-up Study** (HPFS), began in 1986 in the US, aims to evaluate a series of hypotheses about men's heath relating nutritional factors to the incidence of serious illnesses, such as cancer, heart disease, and other vascular diseases. HPFS is all-male study and is designed to complement the all-female Nurses' Health Study. HPFS enrolled 51,529 men aged 40 to 75 at the baseline. The men have been followed with biennial questionnaires to obtain updated demographic, lifestyle, and other information, mirrored the NHS. We used a case-control study of coronary heart study nested within HPFS (HPFS_CHD).[12] The study was reviewed by the Internal Review Board and approved by the Human Subjects Committee at Harvard T.H.Chan School of Public Health.

The **Invecchiare in Chianti, aging in the Chianti Area Study** (InChianti) is a prospective population-based epidemiological study of elder persons living in the Chianti geographical area in Italy. The study was initiated in 1998 and included a representative sample of the older population with 1,453 individuals at the baseline. Fasting blood samples were collected after the baseline visit. The participants were followed up every three years by experienced interviewers.[13] Genotyping was completed using the Illumina Infinium HumanHap 550 chip.[14] The Italian National Research Council on Aging Ethical Committee ratified the entire study protocol.

The **Cooperative Health Research in the Region Augsburg** (KORA) is a regional research platform for population-based surveys and subsequent follow-up studies in the fields of epidemiology, health economics, and health care research established in 1996 in Germany, with a special focus on the

determination of risk factors and treatment options on myocardial infarction rates in populations.[15] For the genome-wide association study, 1,805 randomly selected participants were genotyped with Affymetrix 6.0 array.[16] The study was reviewed and approved by the Ethics Committee of the Bavarian Medical Association.

The **Leiden Longevity Study** (LLS) was designed to identify genetic and phenotypic markers related to familial longevity. A total of 421 families were recruited, consisting of long-lived white siblings, offspring and the offspring's partners. Families were only included if at least 2 long-lived siblings were still alive and met the age criteria (≥ 89 years for men; ≥ 91 years for women). There was no selection based on health conditions or demographic characteristics.[17] Genome-wide SNP data were obtained by use Illumina Infinium HD Human660W-Quad BeadChips and Illumina OmniExpress BeadChips.[18] The study protocols were reviewed and approved by the Medical Ethical Committee of the Leiden University Medical Center.

The **Ludwigshafen Risk and Cardiovascular Health Study** (LURIC) recruited white patients hospitalized for coronary angiography between 1997 and 2004 at a tertiary care center in Southwestern Germany. A standardized personal and family history questionnaire and an extensive laboratory work-up was obtained from all individuals. Fasting venous blood was sampled for the determinations of genotypes and biochemical phenotypes.[19] The present study included data from 2,864 participants with Affymetrix 6.0 genotyping data available.[20] The study protocols were reviewed and approved by the ethics committee at the Landesärztekammer Rheinland-Pfalz.

The **Multi-Ethnic Study of Atherosclerosis** (MESA) was initiated in 1999 to investigate the prevalence, correlates, and progression of subclinical cardiovascular disease in a population-based sample of ~6000 men and women aged 45-84 years recruited from six field centers across the US. Thirty-eight percent of the recruited participants are white, 28% African-American, 22% Hispanic, and 12% Asian, predominantly of Chinese descent. Participants were recruited from six field centers across the United States. Baseline measurements included extensive aspects of cardiovascular disease related phenotypes, sociodemographic factors, medication use and psychosocial factors. Blood samples were assayed and DNA were extracted for genetic studies. Participants are followed-up up with a clinical exam every two years (six exams have been completed) and a yearly phone call to assess cardiovascular events. Genotype data were generated from the Affymetrix 6.0 array.[21] The tenets of the Declaration of Helsinki were followed and institutional review board approval was granted at all MESA sites. Written informed consent was obtained from each participant. We included only MESA participants of Caucasian-ancestry in our current study. The study protocols were reviewed and approved by the Institutional Review Board of each of the six field centers.

The **Nijmegen Biomedical Study** (NBS) is a population-based epidemiological survey conducted in the eastern part of the Netherlands. Randomly selected inhabitants from Nijmegen were invited to participate in the study by receiving a postal questionnaire on lifestyle and medical history. Blood samples were collected at the baseline from individuals who consented to participate in the study. The present study included data from 2,610 participants with Illumina HumanHap300 BeadChip genotyping data was available.[22] The study protocols were reviewed and approved by the Institutional Review Board of the Radboud University Medical Center in Nijmegen.

The **Nurses' Health Study** (NHS) was established in 1976, when 121,700 female US registered nurses, 30-55 years of age and residing in eleven of the larger US states completed an initial questionnaire.[23] During 1989-1990, blood samples were collected from 32,826 women. In 1989, the NHSII was established when 116,671 female registered nurses aged 25-44 years completed an initial questionnaire. During 1996-1999, blood samples were collected from 29,611 women in NHSII.[24] All women have been followed by mailed questionnaires every two years to update exposure information and ascertain non-fatal incident diseases. The follow-up rates for each biennial cycle have consistently been ~90% for both cohorts. We used data of two case-control studies, one for breast cancer (**Cancer Genetic Markers of Susceptibility**, CGEMS, NHS_BRCA)[25] (individuals genotyped on Illumina 550K) and one for type 2 diabetes (T2D, NHS_T2D)[4] (individuals genotyped using Affymetrix 6.0 platform), nested within the NHS. The study was reviewed by the Internal Review Board and approved by the Human Subjects Committee at Partners Healthcare System, and the Brigham and Women's Hospital in Boston, Massachusetts.

The **Orkney Complex Disease Study** (ORCADES) is a cross-sectional genetic epidemiology study based in an isolated population in the north of Scotland, UK, concerned with identifying the genes and variants influencing the risk of common, complex diseases. Individuals must have at least two Orcadian grandparents to be eligible to participate, and recruitment of 2,080 subject was completed in 2011. Up to 500 disease-related phenotypes, high density Illumina genotyping and a biobank of DNA, plasma, serum, and whole blood on all 2080 subjects are available.[26] The study was approved by Local Research Ethics Committees of NHS Orkney and NHS Grampian.

The **Prostate, Lung, Colorectal and Ovarian Cancer Screening Trial** (PLCO) is an ongoing large population-based randomized trial evaluating screening programs for these cancers. PLCO enrolled a total of 76,685 men and 78,216 women, ages 55 to 74, to randomly assigned intervention arm or control arm. The participants completed questionnaires at both baseline and follow-up visits on a broad range of questions including their dietary, health status, etc. Among participants in the screening arm, serum samples are collected and stored for all study years. Plasma, red blood cells and buffy coat DNA fractions are stored for some years. Buccal cells were collected from participants in the control arm of the trial and served primary as a DNA resource.[27] Samples were genotyped with the HumanHap550, HumanHap300 and HumanHap240, Illumina.[28] The study protocols were reviewed and approved by the National Cancer Institute Special Institutional Review Board (SSIRB).

The **Prospective Study of Pravastatin in the Elderly at Risk** (PROSPER) was designed to investigate the benefits of treatment with pravastatin (a cholesterol-lowering drugs) in elderly patients. After screening, a total of 5,804 eligible subjects aged 70 to 82 years were recruited in Scotland, Ireland and the Netherlands between 1997 and 1999. A detailed medical history was taken, and a fasting venous blood sample was drawn for each subject.[29] The genotyping was conducted using the Illumina 660-Quad beadchips.[30] The study protocols were reviewed and approved by the Institutional Ethics Review Boards of centers of Cork University (Ireland), Glasgow University (Scotland) and Leiden University Medical Center (the Netherlands).

The **Study of Health in Pomerania** (SHIP), a population-based project conducted in Northeast Germany, aims to assess prevalence and incidence of common risk factors, subclinical disorders and clinical diseases, and to investigate associations and interactions among them. The SHIP cohort included 4,308 individuals at the baseline and an additional 3,300 individuals after 5 years. The final sample, completed data collection in 2001, included 4310 males and females aged 20 to 79 years, equivalent to a participation rate of 69%.[31,32] A simple medical examination, and a self-administrated questionnaire were conducted in each individual. Blood and urine samples were obtained according to standardized procedures. The genotyping was performed using the Human SNP 6.0 Array (Affymetrix).[33] The study protocols were reviewed and approved by the Institutional Ethics Review Boards of the University of Greifswald.

The **Scottish Colorectal Cancer Study** (SOCCS) is a case-control study of colorectal cancer in Scotland, UK, recruiting subjects prospectively on a population-wide basis between 1996 and 2006. The study included incident colorectal cancer cases, and 3,123 adult cancer-free controls, identified from the Community Health Index in Scotland as being aged within 5 years of their matched case, of the same sex and living in the same area.[34] Genotyping was conducted using custom Illumina Infinium arrays.[35] Ethical approval was obtained from Multi-Center Research Ethics Committee for Scotland, 18 Local Research Ethics committees, 18 Caldicott guardians and 16 NHS Trust management committees.

The **Cardiovascular Risk in Young Finns Study** (LASERI, or YFI) is one of the largest follow-up studies into cardiovascular risk from childhood to adulthood launched in the late 1970s in Finland. The baseline study enrolled 3,596 randomly chosen subjects aged from 3 to 18. After that, several follow-up surveys have been conducted up till 2011/12. Examinations included comprehensive data collection using questionnaires, physical measurements and blood tests.[36] In the present study, 1,984 randomly selected individuals were genotyped using a custom-built Illumine Human 670k BeadChip.[37] The study protocols were reviewed and approved by Institutional Review Boards of Ethics Committee of the Hospital District of Southwest Finland.

The **Prospective Investigation of the Vasculature in Uppsala Seniors** (PIVUS) was started in 2001, with the primary aim to investigate the predictive power of different measurements of endothelial function and arterial compliance in a random sample of 1,016 subjects aged 70 living in the community of Uppsala, Sweden. The participants were asked to answer a questionnaire about their medical history, smoking habits and regular medication. A

fasting blood was sampled.[38] Genotyping was performed using the Illumina's Golden Gate assay[39] and the CardioMetabochip.[40] The study protocols were reviewed and approved by Regional Ethical Review Board in Uppsala.

The **Uppsala Longitudinal Study of Adult Men** (ULSAM) is a longitudinal, epidemiologic study based on all available men, born between 1920 and 1924 in Uppsala County, Sweden. Subjects were invited to participate in a health examination at age 50, and were investigated 6 times at age 60, 70, 77, 82, 88 and 93 years of age.[41] Blood samples were obtained from men at examination at 70 years of age, or 77 years of age. Genotyping was performed using the Illumina's Golden Gate assay[39] and the CardioMetabochip.[42] The study protocols were reviewed and approved by Regional Ethical Review Board in Uppsala.

Replication cohort descriptions

EPIC-InterAct study (n=18,078) EPIC-InterAct is a case-cohort study nested within the European Prospective Investigation into Cancer and Nutrition study (EPIC), including 12,403 incident type 2 diabetes cases verified from 340,234 participants across eight European countries (France, Italy, Spain, UK, Netherlands, Germany, Sweden and Denmark), and a subcohort of 16,154 members (with 778 verified incident type 2 diabetes cases as feature of case-cohort design) randomly selected from these participants.[43]

Genome-wide genotyping in the InterAct study was performed among 23,019 participants with two chips: Illumina HumanCoreExome chip (n=13,725) and Illumina 660W-Quad BeadChip (n=9,294). Genotype imputation was performed to the Haplotype Reference Consortium (HRC) reference panel using IMPUTE v2 software. A total of 18,078 participants with both genotype and baseline plasma 25(OH)D_3 data were included in the analysis. The GWAS in EPIC-InterAct was conducted stratified by the subcohort status (subcohort and non-subcohort) and GWAS chips used respectively. Therefore, four GWAS were performed in InterAct: CoreExomeChip in subcohort (n=6,932), 660W chip in subcohort (n=3,844), CoreExomeChip in non-subcohort (n=4,114), and 660W chip in non-subcohort (n=3,188).

EPIC-CVD study (n=12,253) EPIC-CVD is a large, prospective, case-cohort study nested within the EPIC study,[44] with a random subcohort of 18,249 participants and 24,557 adults who later developed CVD during the follow-up, stratified by centre and selected from the EPIC participants with a stored blood sample available. EPIC-CVD involved participants from ten European countries (France, Italy, Spain, UK, Netherlands, Germany, Sweden, Denmark, Norway and Greece). Genome-wide genotyping in EPIC-CVD was conducted using the Illumina Human Exome v1.1 SNP array. Genotype imputation was performed using the Haplotype Reference Consortium (HRC) reference panel and IMPUTE v2 software. As EPIC-CVD shared the random subcohort with the EPIC-InterAct for eight participating countries, we excluded any overlap samples with InterAct for the GWAS. GWAS was separately performed for subcohort (n=887) and non-subcohort (n=11,366) in EPIC-CVD, with a total of 12,253 participants included in the analyses.

EPIC-Norfolk study (n=10,231) EPIC-Norfolk is one of the two UK constituents of the EPIC study. Between 1993 and 1997, a total of 25,639 men and women aged 40-79 were recruited as baseline of EPIC-Norfolk study.[45,46] EPIC-Norfolk participants were followed up between 1997 and 2000 and between 2004 and 2011, and the 20 years' follow-up is still going on currently. Plasma 25(OH)D used in the present study was measured from the blood samples collected between 1997 and 2000.[46] Genome-wide genotyping in EPIC-Norfolk was conducted among 21,044 participants using Affymetrix UK Biobank Axiom Array. Genotype imputation was performed using the Haplotype Reference Consortium (HRC) reference panel and IMPUTE v2 software. EPIC-Norfolk participants, who were part of the EPIC-InterAct or EPIC-CVD study, were excluded to avoid duplication. Finally, 10,231 participants with both genotyping and plasma 25(OH)D_3 data were included for the GWAS.

The study was approved by the local ethics committees in the EPIC participating countries and the International Review Board of the International Agency for Research on Cancer.

Supplementary Table 1. Study characteristics of the individual cohort included in the meta-GWAS.

Study name	Study design	In 2010 Lancet paper	In current meta-GWAS	In G-by-E interaction analysis	Number of Subjects (maximum or common)	Number of genotyped/ imputed SNPs (millions)	Genotyping platform	Vitamin D measurements
**Previous cohorts by 2010**								
1958 British Birth Cohort (1958BC)	population cohort	Yes, as discovery	Yes	Yes	Type 1 Diabetes Genetics Consortium: 2393; Wellcome Trust Case Control Consortium: 2592; total: 4985	~2.5m	Affymetrix 500K or Illumina 550K	ELISA
Cardiovascular Health Study (CHS)	population cohort	Yes, as in-silico replication	Yes		1791	~2.2m	Illumina 317K CNV chip	high-performance liquid chromatography-tandem mass spectrum
Framingham Heart Study (FHS)	population cohort	Yes, as discovery	Yes	Yes	5654	~2.5m	Affymetrix 500K and MIPS 50K	RIA
Gothenburg Osteoporosis and Obesity Determinants Study (GOOD)	population cohort	Yes, as in-silico replication	Yes		921	~2.5m	Human610-Quadv1	RIA
Health, Aging, and Body Composition Study (Health ABC)	population cohort	Yes, as in-silico replication	Yes	Yes	1558	~2.5m	Illumina 1M	RIA
The Study of Indiana Women (Indiana)	population cohort	Yes, as in-silico replication	Yes		567	~2.2m	Human610-Quadv1	high-performance liquid chromatography-tandem mass spectrum
North Finland Birth Cohort 1966 (NFBC)	population cohort	Yes, as in-silico replication	Yes	Yes	4604	~2.4m	Illumina Infinium cnvDuo array	high-performance liquid chromatography-tandem mass spectrum
Old Order Amish Study (OOA)	population cohort	Yes, as discovery	Yes		330	~2.3m	Affymetrix 500K and 100K	RIA
The Rotterdam Study (RS)	population cohort	Yes, as discovery	Yes	Yes	1237	~2.5m	HumanHap550K	RIA
TwinsUK	population cohort	Yes, as discovery	Yes		TwinsUK I: 2152; TwinsUK II:2983; total: 5135	~2.4m	Illumina 317K or 610K	RIA
**New cohorts since 2010**								

Alpha-Tocopherol, Beta-Carotene Cancer Prevention Study (ATBC)	case-control study	No	Yes	Yes	865 cases, 507 controls; total: 1372	~2.2m	Illumina 550K (or higher version) platform	RIA and chemiluminescence immunoassay
Prostate, Lung, Colorectal, and Ovarian Cancer Screening Trial (PLCO)	case-control study	No	Yes	Yes	728 cases, 587 controls; total: 1315	~2.2m	Illumina 240K, 300K, and 550K platform	RIA
Atherosclerosis Risk In Communities (ARIC)	population cohort	No	Yes	Yes	8124	~2.5m	Affymetrix 6.0 array	liquid chromatography-tandem high-sensitivity mass spectrometry
AtheroGene	population cohort	No	Yes		1062	~0.6m	Illumina array	RIA
B-Vitamins for the Prevention Of Osteoporotic Fractures (BPROOF)	population cohort	No	Yes	Yes	2525	~2.5m	IlluminaOmniExpression array	liquid chromatography-tandem mass spectrometry
Epidemiology of Diabetes Interventions and Complications (EDIC)	population cohort	No	Yes		1094	~1.7m	Human 1M BeadChip	liquid chromatography-tandem mass spectrometry
Case-Control Study for Metabolic Syndrome (GENMETS)	case-control study	No	Yes		820 cases, 821 controls; total: 1641	~1.2m	Illumina 610K	RIA
The Helsinki Birth Cohort Study (HBCS)	population cohort	No	Yes		917	~2.4m	Illumina 670K	high-performance liquid chromatography
Coronary Heart Disease Case-Control Study nested within NHS and HPFS (HPFS_CHD)	case-control study	No	Yes	Yes	413 cases, 832 controls; total: 1245	~2.5m	Illumina 550K (or higher version) platform	RIA
The Invecchiare in Chianti Study (InChianti)	population cohort	No	Yes		1094	~2.5m	Illumina Infinium HumanHap 550 chip	enzyme immunoassay (OCTEIA 25-hydroxyvitamin D kit)
Cooperative Health Research in the Region Augsburg (KORA)	population cohort	No	Yes		1805	~0.8m	Affymetrix 6.0 array	enhanced chemiluminescence immunoassay
Leiden Longevity Study (LLS)	population cohort	No	Yes		2265	~1.9m	Illumina Infinium HD Human660W-Quad and Illumina OmniExpress BeadChips	electrochemiluminescence immunoassays
Ludwigshafen Risk and Cardiovascular Health Study (LURIC)	population cohort	No	Yes		LURIC1: 769; LURIC2: 2077; total: 2846	~2.3m, ~3.6m	Affymetrix 6.0 array	RIA
Multi-Ethnic Study of Atherosclerosis (MESA)	population cohort	No	Yes	Yes	2240	~2.7m	Affymetrix 6.0 array	high-performance HPLC–tandem mass spectrometry
Nijmegen Biomedische Studie (NBS)	population cohort	No	Yes		2610	~2.5m	Illumina HumanHap300	RIA

Breast Cancer Case-Control Study nested within NHS (NHS_BRCA)	case-control study	No	Yes	Yes	440 cases, 430 controls; total: 870	~2.5m	Illumina 550K (or higher version) platform	RIA
Type II diabetes Case-Control Study nested within NHS (NHS_T2D)	case-control study	No	Yes	Yes	361 cases, 363 controls; total: 724	~2.5m	Affymetrix 6.0 array	RIA
The Orkney Complex Disease Study (ORCADES)	population cohort	No	Yes		847	~2.1m	Illumina HumanHap300	liquid chromatography-tandem mass spectrometry
Prospective Study of Pravastatin in the Elderly at Risk (PROSPER)	population cohort	No	Yes		4871	~2.4m	Illumina 660-Quad BeadChip	electrochemiluminescence immunoassays
More samples from the Rotterdam Study (RS)	population cohort	No	Yes	Yes, RS III	RSI: 3322; RSII: 2038; RS III: 2953; total: 8313	~2.5m	HumanHap550K	RIA
The Study of Health in Pomerania (SHIP)	population cohort	No	Yes		1655	~2.7m	Affymetrix 500K array	IDS-iSYS 25-Hydroxy Vitamin D assay
The Scottish Colorectal Cancer Study (SOCCS)	case-control study	No	Yes	Yes	449 cases, 716 controls; total: 1165	~2.5m	custom Illumina Infinium array	liquid chromatography-tandem mass spectrometry
Cardiovascular risk in Young Finns Study (YFS)	population cohort	No	Yes	Yes	1984	~1.2m	Illumina Human 670K BeadChip	RIA
Prospective Investigation of the Vasculature in Uppsala Seniors (PIVUS)	population cohort	No	No	Yes	989	~2.7m	Illumina's Golden Gate assay and CardioMetabochip	chemiluminescence immunoassay
Uppsala Longitudinal Study of Adult Men (ULSAM)	population cohort	No	No	Yes	1124	~4.5m	Illumina's Golden Gate assay and CardioMetabochip	high-performance liquid chromatography-tandem mass spectrum
**Sample size (maximum or common)**			79366	41981				

ELISA: Enzyme-linked immunosorbent assay; RIA: radioimmunoassay;

Supplementary Table 2. Results from conditional and joint genome-wide association analysis for the six GWAS-identified loci.

Gene	SNP	Chromosome: Position	Effect Allele	Allele Frequency	Effect (Beta)	Standar d Error	P-value
GC	rs7662029	4:69996501	A	0.51	0.012	0.0021	1.07E-08
	rs1385986	4:72111975	A	0.03	-0.035	0.0059	2.46E-09
	rs17383291	4:72824274	T	0.90	0.051	0.0055	1.17E-20
	rs3755967*	4:72828262	T	0.28	-0.0667	0.0031	4.98E-101
	rs222040	4:72835796	A	0.56	0.028	0.003	9.08E-20
	rs1474198	4:72987876	T	0.55	0.015	0.0022	1.46E-12
CYP2R1	rs11023314	11:14701509	A	0.96	-0.048	0.0061	3.73E-15
	rs1007392	11:14731167	A	0.56	0.039	0.0035	1.71E-28
	rs1542291	11:14762842	T	0.40	-0.062	0.0041	6.05E-53
	rs2167453	11:14822242	T	0.90	0.054	0.0043	3.73E-36
	rs2060793	11:14871886	A	0.39	0.057	0.0038	2.20E-51
NADSYN1/ DHCR7	rs12785878*	11:70845097	T	0.75	0.036	0.0022	5.70E-61
AMDHD1	rs10745742*	12:94882660	T	0.40	0.013	0.0023	7.73E-09
	rs7308827	12:94906775	A	0.82	0.015	0.0027	1.86E-08
SEC23A	rs8018720*	14:38625936	C	0.82	-0.017	0.0029	6.97E-09
CYP24A1	rs17216707*	20:52165769	T	0.78	0.026	0.0027	2.17E-22

For the six GWAS-identified loci, we further tested whether any other SNPs, in addition to the top SNP, were significantly associated with the serum 25-hydroxyvitamin D concentrations by using COJO analysis implemented in GCTA. We found multiple independently associated variants at loci *GC*, *CYP2R1*, and *AMDHD1*. *: GWAS-identified lead SNP in each locus. Beta coefficients, standard errors, and P-values were from the joint analysis.

Supplementary Table 3. Significant SNPs from the marginal genetic effect screening performed in a subset of cohorts used for the SNP-by-dietary vitamin D interaction.

SNP	Gene	Effect Allele	Allele Frequency	Effect	P-value
rs2282679	*GC*	T	0.74	0.087	2.40E-189
rs10741657	*CYP2R*	A	0.41	0.034	1.10E-36
rs4944062	*NADSYN1/ DHCR7*	T	0.70	0.035	4.30E-33
rs10745742	*AMDHD1*	T	0.39	0.016	5.70E-09
rs17216707	*CYP24A1*	T	0.79	0.028	1.50E-15
rs8018720	*SEC23A*	C	0.84	-0.015	8.94E-05

Supplementary Table 4. Enrichment estimates for the 24 main annotations, the 500bp windows around each of the 24 main annotations, and the 100-bp windows around ChIP-seq peaks.

	24 main annotations				24 main annotations + 500bp				24 main annotation Peak			
**Category**	**Prop. of SNPs (%)**	**Prop. of h^2 (%)**	**Enrichment (SE)**	**Enrichment P-values**	**Prop. of SNPs (%)**	**Prop. of h^2 (%)**	**Enrichment (SE)**	**Enrichment P-values**	**Prop. of SNPs (%)**	**Prop. of h^2 (%)**	**Enrichment (SE)**	**Enrichment P-values**
Weak Enhancer	2.11	42.28	20.04(7.79)	**0.02**	8.9	12.2	1.37(1.73)	0.83				
Conserved region	2.61	35.9	13.77(6.23)	**0.03**	33.25	47.68	1.43(0.58)	0.45				
Promoter Flanking	0.84	8.94	10.61(9.72)	0.32	3.35	24.23	7.24(3.48)	0.06				
TSS	1.82	19.14	10.51(5.67)	0.087	3.48	35.25	10.12(3.69)	**0.01**				
Fetal DHS	8.48	78.55	9.27(3.52)	**0.02**	28.5	59.15	2.08(1.08)	0.31				
Enhancer	6.33	44.26	6.99(3.03)	0.065	15.39	39.29	2.55(1.23)	0.2				
TFBS	13.25	75.04	5.67(2.36)	**0.048**	34.34	80.63	2.35(0.76)	0.075				
DHS	16.78	93.17	5.55(2.47)	0.056	49.88	68.84	1.38(0.56)	0.5	11.18	51.53	4.61(3.09)	0.23
H3K27ac (PGC)	26.95	112.36	4.17(0.96)	**8.00E-04**	33.6	86.14	2.56(0.54)	**0.0043**				
Promoter	3.12	11.65	3.74(4.14)	0.51	3.86	14.17	3.67(2.58)	0.29				
Fantom5 Enhancer	0.43	1.55	3.58(16.91)	0.88	1.91	4.99	2.62(5.03)	0.74				
H3K9ac	12.61	42.69	3.39(1.32)	0.075	23.06	56.86	2.47(0.72)	**0.039**	3.88	11.25	2.9(5.41)	0.72
Coding	1.47	4.92	3.35(6.84)	0.73	6.46	13.25	2.05(1.59)	0.51				
H3K4me1	42.66	121.13	2.84(0.6)	**0.0044**	60.92	109.12	1.79(0.24)	**0.0019**	17.13	49.19	2.87(1.74)	0.29
H3K4me3	13.33	33.31	2.5(1.5)	0.33	25.55	85.2	3.33(0.82)	**0.0045**	4.18	-5.01	-1.2(4.98)	0.66
3'UTR	1.11	2.26	2.05(6.53)	0.87	2.69	4.56	1.69(3.08)	0.82				
Super Enhancer	16.84	32.48	1.93(0.45)	**0.043**	17.16	30.73	1.79(0.48)	0.088				
H3K27ac	39.12	75	1.92(0.36)	**0.012**	42.26	56.55	1.34(0.35)	0.33				
Intron	38.75	55.73	1.44(0.3)	0.14	39.71	57.56	1.45(0.23)	0.058				
Repressed region	46.12	61.83	1.34(0.61)	0.58	71.91	39.46	0.55(0.16)	**0.006**				
CTCF	2.38	1.94	0.82(5.77)	0.97	7.11	15.94	2.24(2.04)	0.54				
DGF	13.76	11.13	0.81(2.55)	0.94	54.15	50.16	0.93(0.52)	0.89				
Transcribed	34.54	3.81	0.11(0.72)	0.22	76.31	55.86	0.73(0.19)	0.16				
5'UTR	0.54	-5.28	-9.74(11.16)	0.32	2.78	-1.95	-0.7(3.17)	0.58				

Black bold font indicates P-values less than 0.05; Red bold font indicates significant P-values after multiple corrections (P<0.05/24). TSS: transcription start sites; TFBS: transcription factor binding sites; DHS: DNase I hypersensitive sites; CTCF: CCCTC-binding factor; DGF: digital genomic footprinting.

Supplementary Table 5. Numbers of individuals and SNPs involved in the 37 traits.

Trait Identifier	Reference	No. of individuals	No. of SNPs	Original files
UKBiobank Age at Menarche	UKBiobank	74944	1187029	
UKBiobank Age at Menopause	UKBiobank	44410	1187051	
UKBiobank Asthma	UKBiobank	145416	1187053	
UKBiobank Diastolic Blood Pressure	UKBiobank	134011	1186980	
UKBiobank Systolic Blood Pressure	UKBiobank	134011	1187025	
UKBiobank BMI	UKBiobank	145209	1186959	
UKBiobank Eczema	UKBiobank	145416	1187034	
UKBiobank Heel T-Score	UKBiobank	141441	1185277	
UKBiobank Height	UKBiobank	145368	1183900	
UKBiobank Hypertension	UKBiobank	145379	1187099	
UKBiobank FEV1FVC ratio	UKBiobank	123935	1186631	
UKBiobank Forced Vital Capacity	UKBiobank	123935	1187014	
UKBiobank Waist Hip Ratio	UKBiobank	145375	1186973	
Alzheimer's Disease	Lambert et al., 2013 Nat Genet	54162	1136071	
Autism Spectrum	PGC Cross-Disorder Group, 2013 Lancet	10263	1173308	http://www.med.unc.edu/pgc/files/resultfiles/pgcasdeuro.gz
Celiac Disease	Dubois et al., 2010 Nat Genet	15283	245449	https://www.immunobase.org/downloads/protected_data/GWAS_Data/
Depressive Symptoms	Okbay et al., 2016 Nat Genet	161460	1115394	http://ssgac.org/documents/DS_Full.txt.gz
Inflammatory Bowel Disease	Jostins et al., 2012 Nature	34652	1078061	http://www.ibdgenetics.org/downloads.html
Multiple Sclerosis	IMS Genetics Consortium, 2011 Nature	27148	227549	https://www.immunobase.org/downloads/protected_data/GWAS_Data/
Neuroticism	Okbay et al., 2016 Nat Genet	170911	1115394	http://ssgac.org/documents/Neuroticism_Full.txt.gz
Primary Biliary Cirrhosis	Cordell et al., 2015 Nat Com	13239	525775	https://www.immunobase.org/downloads/protected_data/GWAS_Data/
Subject Well Being	Okbay et al., 2016 Nat Genet	298420	477900	http://ssgac.org/documents/SWB_Full.txt.gz
Systemic Lupus Erythematosus	Bentham et al., 2015 Nat Genet	14267	654940	https://www.immunobase.org/downloads/protected_data/GWAS_Data/
Anorexia	Boraska et al., 2014 Mol Psych	32143	931185	http://www.med.unc.edu/pgc/downloads/
Bipolar Disorder	BIP Working Group of the PGC, 2011 Nat Genet	16731	750636	http://www.med.unc.edu/pgc/downloads/
Coronary Artery Disease	Schunkert et al., 2011 Nat Genet	77209	925224	http://www.cardiogramplusc4d.org/
Crohn's Disease	Jostins et al., 2012 Nature	20883	1051515	http://www.ibdgenetics.org/downloads.html
Ever Smoked	TAG Consortium, 2010 Nat Genet	74035	1060318	http://www.med.unc.edu/pgc/downloads/
Fasting Glucose	Manning et. al., 2012 Nat Genet	46186	1115625	http://www.magicinvestigators.org/downloads/
High-density Lipoprotein	Teslovich et al., 2010 Nature	97749	1019273	http://www.broadinstitute.org/mpg/pubs/lipids2010/
Low-density Lipoprotein	Teslovich et al., 2010 Nature	93354	1017974	http://www.broadinstitute.org/mpg/pubs/lipids2010/
Rheumatoid Arthritis	Okada et al., 2014 Nature	37681	562682	http://plaza.umin.ac.jp/yokada/datasource/software.html
Schizophrenia	SCZ Working Group of the PGC, 2014 Nature	70100	1083015	http://www.med.unc.edu/pgc/downloads/
Triglycerides	Teslovich et al., 2010 Nature	94460	1017799	http://www.broadinstitute.org/mpg/pubs/lipids2010/
Type 2 Diabetes	Morris et al., 2012 Nat Genet	60786	968539	http://www.diagram-consortium.org/
Ulcerative Colitis	Jostins et al., 2012 Nature	27432	1076835	http://www.ibdgenetics.org/downloads.html
Age-related Macular Degeneration	Fritsche et al. (2015) Nature Genetics	33976	12000000	http://amdgenetics.org/

Supplementary Table 6. Directional genetic correlation analysis between 25-hydroxyvitamin D and traits.

Phenotype1	Phenotype2	RHO1	RHO2	FZ1	FZ2	SE1	SE2	Likelihood 1_cause_2	Likelihood 2_cause_1	AIC best causal model	AIC non-causal model	ratio
vitamin D	HDL	1.00	0.14	18.37	0.14	0.71	0.11	-0.16	-336.66	2.32	649.67	**2.68E-141**
vitamin D	Crohn's Disease	-0.26	0.44	-0.26	0.47	0.58	0.24	-1.78	0.02	1.95	3.13	0.55
vitamin D	Systemic Lupus	-0.38	0.43	-0.40	0.46	0.58	0.27	-1.45	-0.20	2.41	3.36	0.62
vitamin D	Rheumatoid Arthritis	-0.80	0.13	-1.10	0.13	0.71	0.27	-0.29	-1.38	2.58	2.99	0.81
vitamin D	Ulcerative Colitis	-0.71	-0.29	-0.90	-0.30	0.58	0.26	-0.60	-1.14	3.21	2.76	1.25
vitamin D	LDL	0.70	0.09	0.87	0.09	0.71	0.12	0.35	-0.12	1.29	0.80	1.28
vitamin D	TG	-0.20	-0.15	-0.20	-0.15	0.71	0.13	-0.10	0.52	0.95	0.29	1.40
vitamin D	Bipolar	0.09	0.80	0.09	1.10	0.58	1.00	-1.89	-1.30	4.60	3.81	1.49
vitamin D	Alzheimer	-0.70	-0.60	-0.87	-0.69	0.71	0.71	-1.63	-1.90	5.25	4.32	1.59
vitamin D	Type2 Diabetes	-0.52	-0.07	-0.58	-0.07	0.58	0.32	-0.16	-0.64	2.33	1.33	1.64
vitamin D	Height	0.49	0.00	0.53	0.00	0.58	0.05	1.79	1.37	-1.59	-2.74	1.78
vitamin D	Inflammatory Bowel Disease	-0.49	0.14	-0.53	0.14	0.58	0.18	0.11	0.01	1.78	0.62	1.78
vitamin D	Body Mass Index	0.14	-0.09	0.14	-0.09	0.58	0.11	0.57	0.85	0.31	-1.07	1.99
vitamin D	Coronary Artery Disease	-0.37	-0.01	-0.39	-0.01	0.58	0.28	-0.01	-0.23	2.01	0.47	2.16
vitamin D	Neuroticism	0.32	-0.31	0.33	-0.32	0.58	0.50	-0.80	-0.76	3.52	1.92	2.22
vitamin D	Years of Education	0.14	-0.01	0.14	-0.01	0.58	0.23	0.18	0.15	1.64	-0.30	2.64
vitamin D	Schizophrenia	0.03	0.00	0.03	0.00	0.58	0.17	0.49	0.49	1.02	-0.98	2.71

Only traits with more than 5 significant GWAS hits were included. For example, smoking has only 2 genome-wide significant loci and were therefore not included in the analysis. Let $Beta_{XX}$ be the vector of effect sizes on vitamin D for the set of variants ascertained through the association study (6 SNPs), and $Beta_{XY}$ be the vector of the effect sizes of these variants on phenotype2. Define $Beta_{YY}$ and $Beta_{YX}$ analogously. RHO1 is the rank correlation between $Beta_{XX}$ and $Beta_{XY}$, RHO2 is the rank correlation between $Beta_{YY}$ and $Beta_{YX}$. FZ: Fisher's Z-transformation of RHO; AIC: Akaike information criterion.

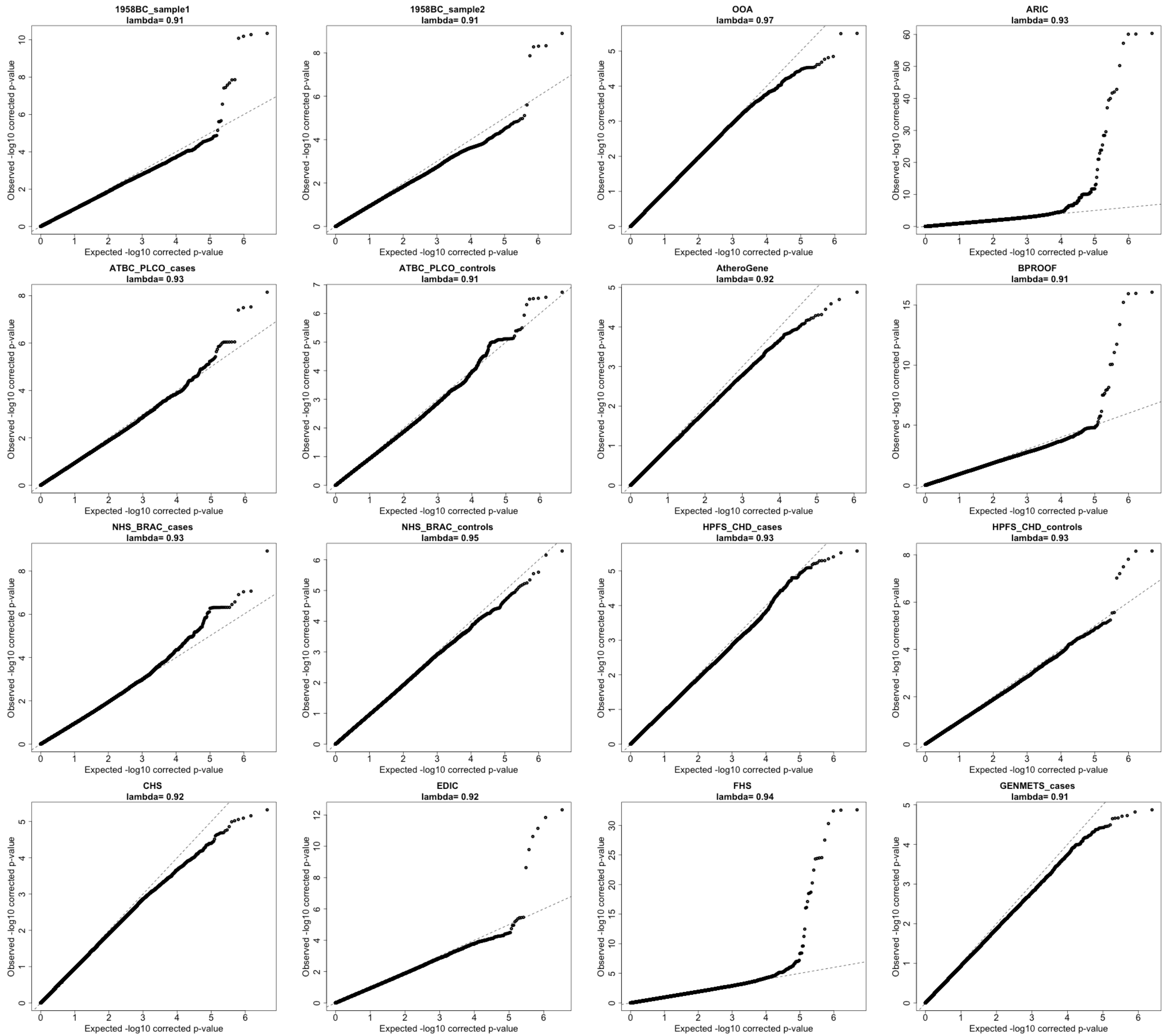
1958BC_sample1
lambda= 0.91
1958BC_sample2
lambda= 0.91
OOA
lambda= 0.97
ARIC
lambda= 0.93
ATBC_PLCO_cases
lambda= 0.93
ATBC_PLCO_controls
lambda= 0.91
AtheroGene
lambda= 0.92
BPROOF
lambda= 0.91
NHS_BRAC_cases
lambda= 0.93
NHS_BRAC_controls
lambda= 0.95
HPFS_CHD_cases
lambda= 0.93
HPFS_CHD_controls
lambda= 0.93
CHS
lambda= 0.92
EDIC
lambda= 0.92
FHS
lambda= 0.94
GENMETS_cases
lambda= 0.91
Observed -log10 corrected p-value
Expected -log10 corrected p-value

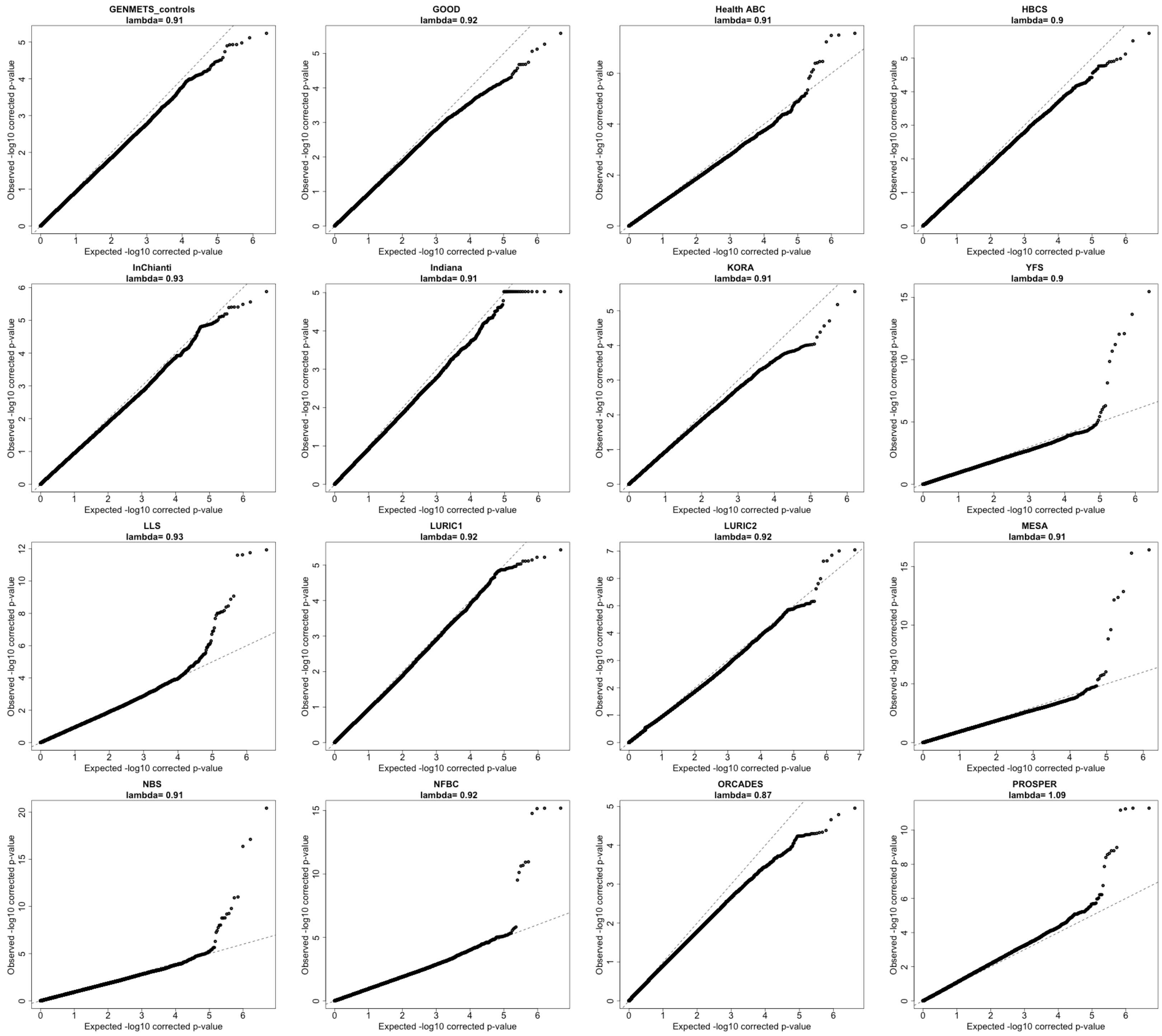
GENMETS_controls
lambda= 0.91
GOOD
lambda= 0.92
Health ABC
lambda= 0.91
HBCS
lambda= 0.9
InChianti
lambda= 0.93
Indiana
lambda= 0.91
KORA
lambda= 0.91
YFS
lambda= 0.9
LLS
lambda= 0.93
LURIC1
lambda= 0.92
LURIC2
lambda= 0.92
MESA
lambda= 0.91
NBS
lambda= 0.91
NFBC
lambda= 0.92
ORCADES
lambda= 0.87
PROSPER
lambda= 1.09
Observed -log10 corrected p-value
Expected -log10 corrected p-value

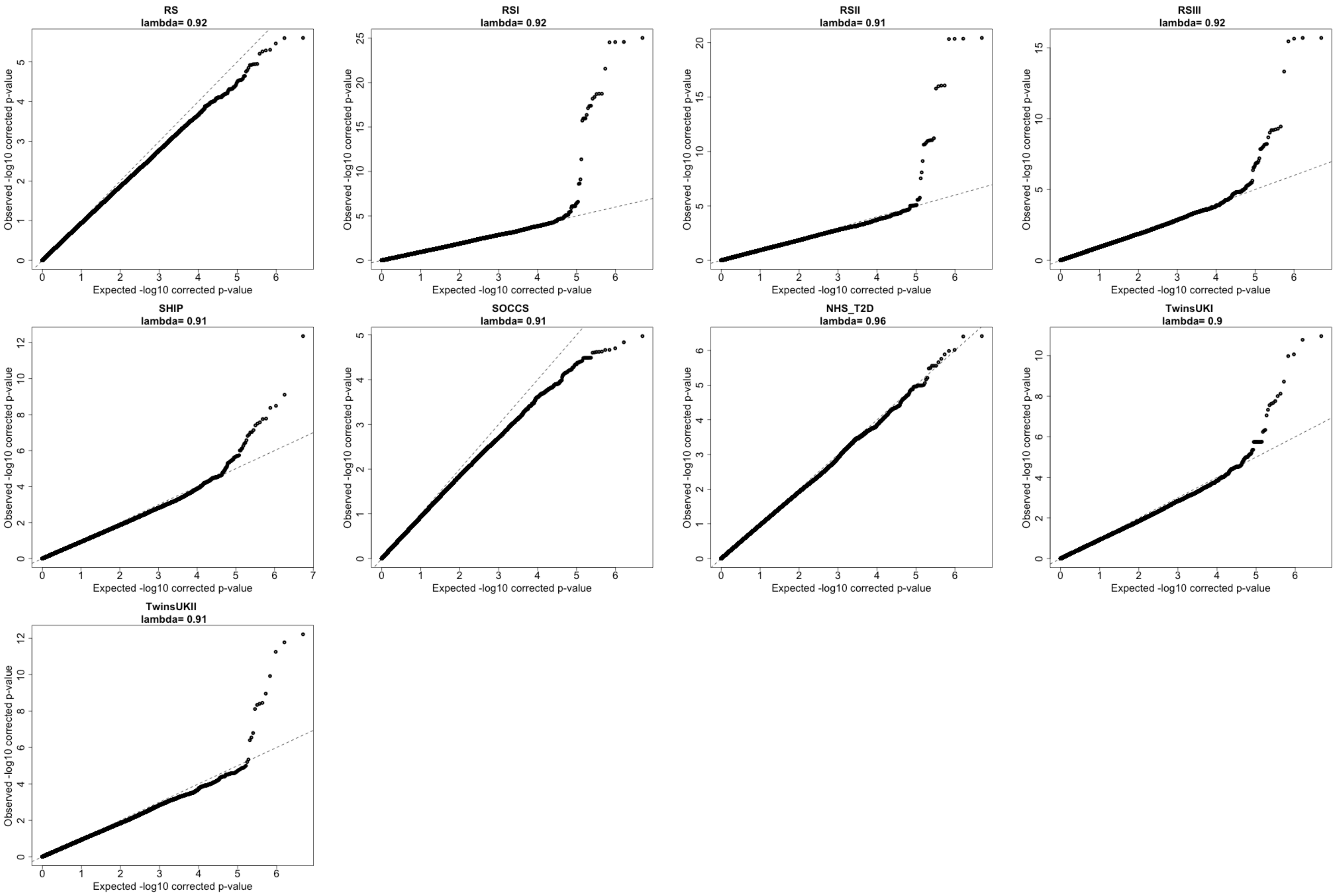

Supplementary Figure 1: QQ-plot for each participating cohort. For contributing studies with a case-control design, we performed QQ-plot separately in cases and controls. Genomic inflation factor (lambda) was estimated and presented above each plot.

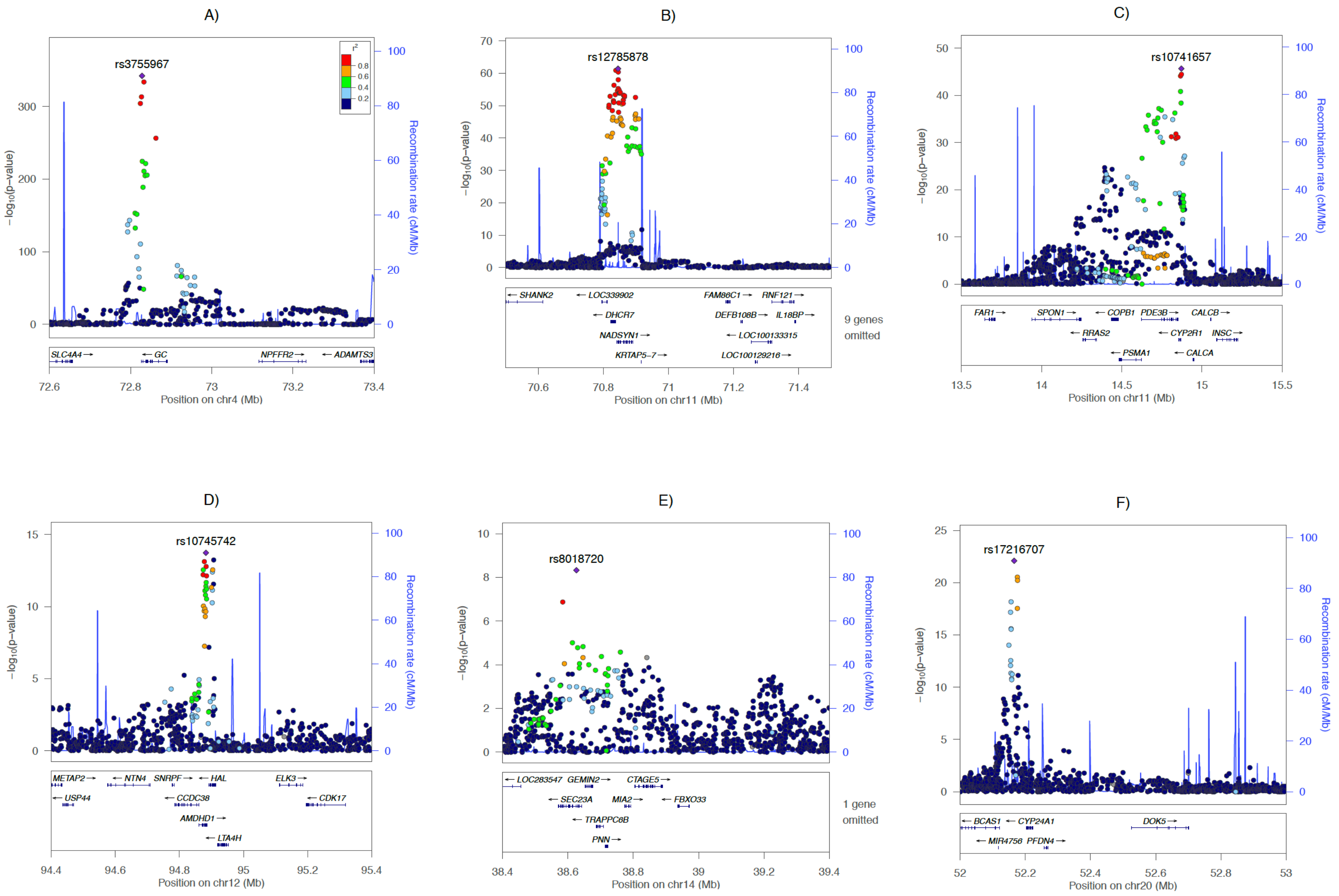

A)
rs3755967
r²
0.8
0.6
0.4
0.2
−log10(p−value)
Recombination rate (cM/Mb)
SLC4A4
GC
NPFFR2
ADAMTS3
Position on chr4 (Mb)
B)
rs12785878
SHANK2
LOC339902
DHCR7
NADSYN1
KRTAP5−7
FAM86C1
RNF121
DEFB108B
IL18BP
LOC100133315
LOC100129216
9 genes omitted
Position on chr11 (Mb)
C)
rs10741657
FAR1
SPON1
COPB1
PDE3B
CALCB
RRAS2
CYP2R1
INSC
PSMA1
CALCA
Position on chr11 (Mb)
D)
rs10745742
METAP2
NTN4
SNRPF
HAL
ELK3
USP44
CCDC38
CDK17
AMDHD1
LTA4H
Position on chr12 (Mb)
E)
rs8018720
LOC283547
GEMIN2
CTAGE5
SEC23A
MIA2
FBXO33
TRAPPC6B
PNN
1 gene omitted
Position on chr14 (Mb)
F)
rs17216707
BCAS1
CYP24A1
DOK5
MIR4756
PFDN4
Position on chr20 (Mb)

Supplementary Figure 2: Regional association plots of the six GWAS-identified loci for 25-hydroxyvitamin D concentration (rs3755967, rs12785878, rs10741657, rs10745742, rs8018720, rs17216707).

A) Regional association plot at rs3755967. Above, the P-values of each SNP are plotted (as $-\log_{10}$ P-values) against their physical position on chromosome 4 (NCBI Build 37). The P-value for rs3755967 is represented by a purple diamond. Estimated recombination rates from the HapMap CEU population show the local LD structure. Inset, the SNP's colors indicate LD with rs3755967 according to a scale from $r^2 = 0$ to $r^2 = 1$ based on pairwise r^2 values from HapMap CEU. Below, gene annotations from the UCSC genome browser. B) Regional association plot at rs12785878. C) Regional association plot at rs10741657. D) Regional association plot at rs10745742. E) Regional association plot at rs8018720. F) Regional association plot at rs17216707.

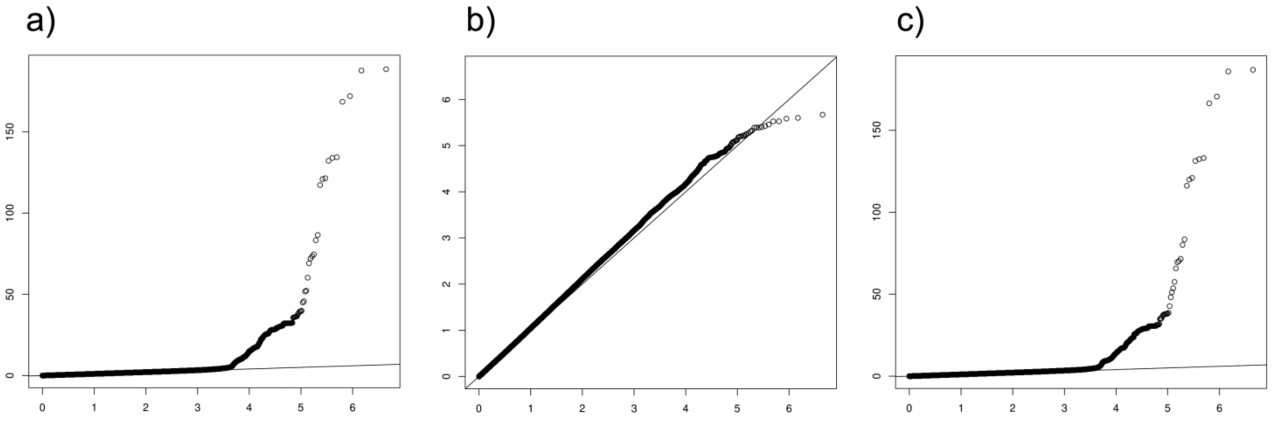

Supplementary Figure 3. QQ-plots from the dietary vitamin D intake analyses.
We performed additional genome-wide association screenings in a subset of 15 cohorts (N = 36,198) having available information on vitamin D intake while considering three models. In model (a), we only included vitamin D as a covariate on top of a marginal genetic effect, in model (b) we performed a 1 degree-of-freedom test for SNP-by-vitamin D intake interaction, and in model (c) we performed a 2 degree-of-freedom test of main SNP effect and SNP-by-vitamin D intake interaction. QQ-plots of the three tests are presented below. Genomic inflation factors equaled 1.03, 1.03, and 1.07, for test (a), (b), and (c), respectively.

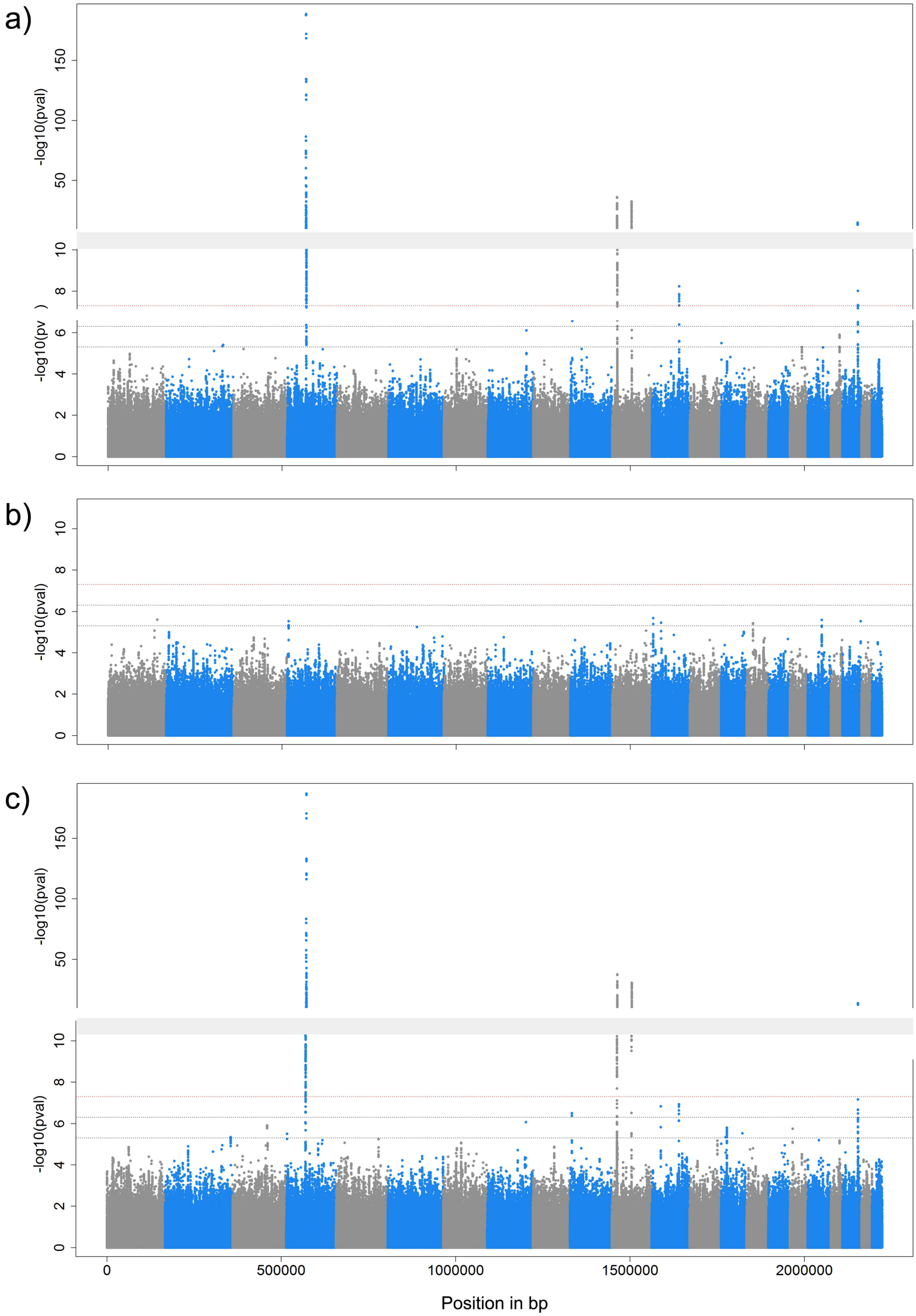
a)
b)
c)
-log10(pval)
0
2
4
6
8
10
50
100
150
0
500000
1000000
1500000
2000000
Position in bp

Supplementary Figure 4. Manhattan plots from the dietary vitamin D intake analyses.
We performed additional genome-wide association screenings in a subset of 15 cohorts (N = 36,198) having available information on dietary vitamin D intake while considering three models. In model (a), we only included vitamin D as a covariate on top of a marginal genetic effect, in model (b) we performed a 1 degree-of-freedom test for SNP-by-vitamin D intake interaction, and in model (c) we performed a 2 degree-of-freedom test of main SNP effect and SNP-by-vitamin D intake interaction. Manhattan plots of the three tests are presented below. Note that for GWAS harboring very low P-value (model a) and c)), plots are separated in 2 parts by a gray bar. Lower part focuses on the standard genome-wide significance threshold of P=5e-08 while the upper part display signal with very low P-values.

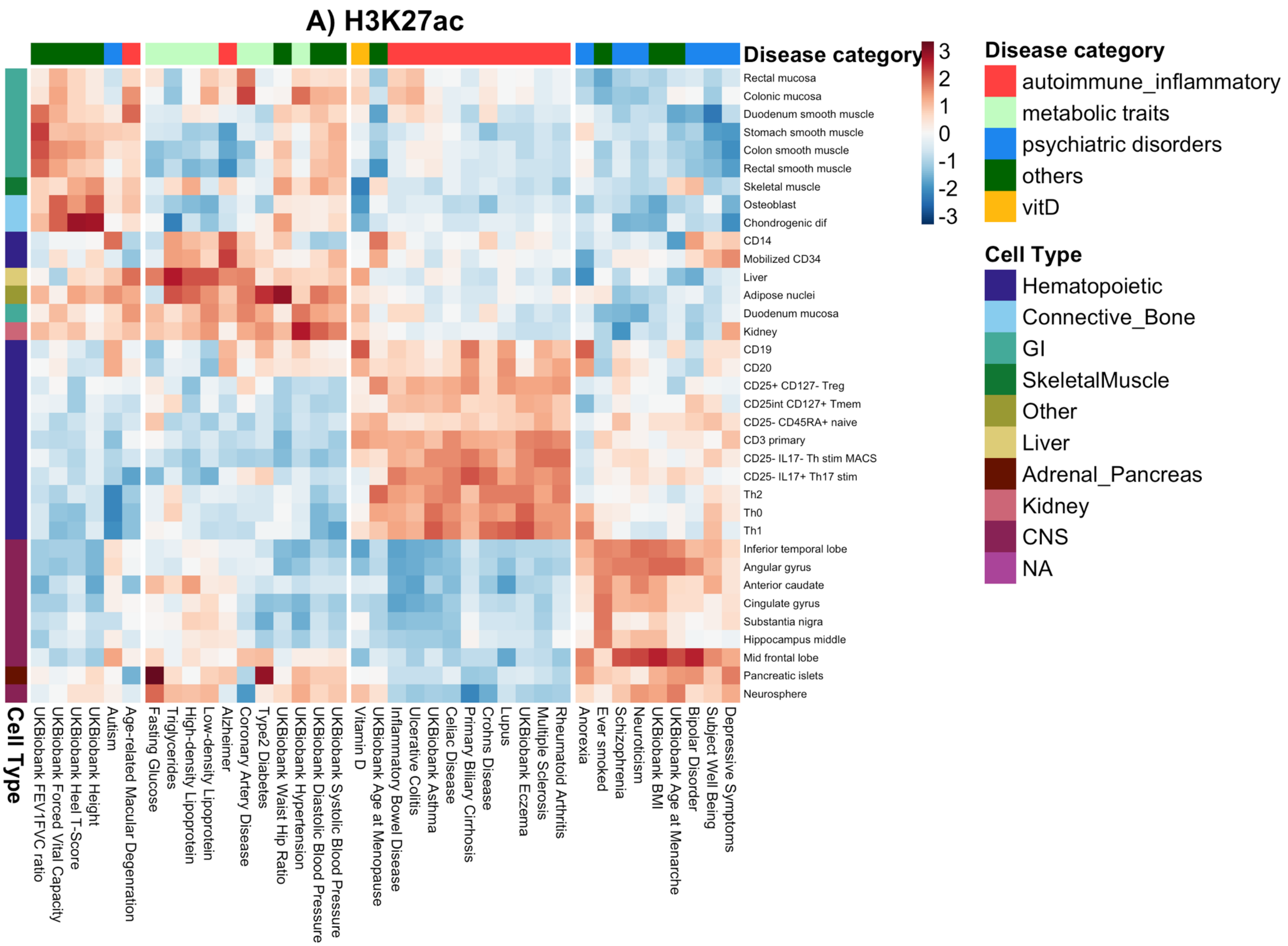
A) H3K27ac
Disease category
3
2
1
0
-1
-2
-3
Disease category
autoimmune_inflammatory
metabolic traits
psychiatric disorders
others
vitD
Cell Type
Hematopoietic
Connective_Bone
GI
SkeletalMuscle
Other
Liver
Adrenal_Pancreas
Kidney
CNS
NA
Rectal mucosa
Colonic mucosa
Duodenum smooth muscle
Stomach smooth muscle
Colon smooth muscle
Rectal smooth muscle
Skeletal muscle
Osteoblast
Chondrogenic dif
CD14
Mobilized CD34
Liver
Adipose nuclei
Duodenum mucosa
Kidney
CD19
CD20
CD25+ CD127- Treg
CD25int CD127+ Tmem
CD25- CD45RA+ naive
CD3 primary
CD25- IL17- Th stim MACS
CD25- IL17+ Th17 stim
Th2
Th0
Th1
Inferior temporal lobe
Angular gyrus
Anterior caudate
Cingulate gyrus
Substantia nigra
Hippocampus middle
Mid frontal lobe
Pancreatic islets
Neurosphere
Cell Type
UKBiobank FEV1FVC ratio
UKBiobank Forced Vital Capacity
UKBiobank Heel T-Score
UKBiobank Height
Autism
Age-related Macular Degeneration
Fasting Glucose
Triglycerides
High-density Lipoprotein
Low-density Lipoprotein
Alzheimer
Coronary Artery Disease
Type2 Diabetes
UKBiobank Waist Hip Ratio
UKBiobank Hypertension
UKBiobank Diastolic Blood Pressure
UKBiobank Systolic Blood Pressure
Vitamin D
UKBiobank Age at Menopause
Inflammatory Bowel Disease
Ulcerative Colitis
UKBiobank Asthma
Celiac Disease
Primary Biliary Cirrhosis
Crohns Disease
Lupus
UKBiobank Eczema
Multiple Sclerosis
Rheumatoid Arthritis
Anorexia
Ever smoked
Schizophrenia
Neuroticism
UKBiobank BMI
UKBiobank Age at Menarche
Bipolar Disorder
Subject Well Being
Depressive Symptoms

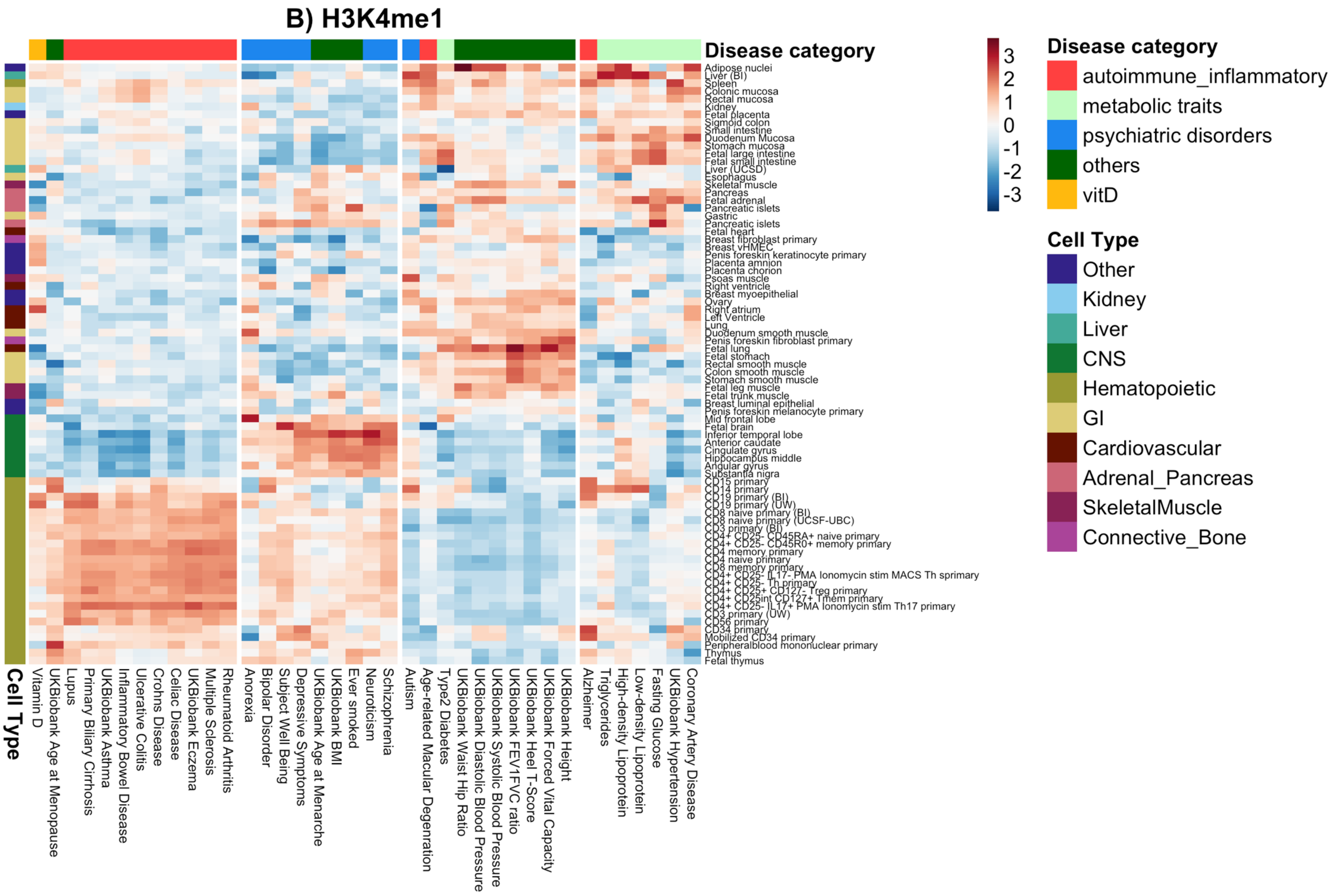
B) H3K4me1
Disease category
autoimmune_inflammatory
metabolic traits
psychiatric disorders
others
vitD
Cell Type
Other
Kidney
Liver
CNS
Hematopoietic
GI
Cardiovascular
Adrenal_Pancreas
SkeletalMuscle
Connective_Bone
3
2
1
0
-1
-2
-3
Adipose nuclei
Liver (BI)
Spleen
Colonic mucosa
Rectal mucosa
Kidney
Fetal placenta
Sigmoid colon
Small intestine
Duodenum Mucosa
Stomach mucosa
Fetal large intestine
Fetal small intestine
Liver (UCSD)
Esophagus
Skeletal muscle
Pancreas
Fetal adrenal
Pancreatic islets
Gastric
Pancreatic islets
Fetal heart
Breast fibroblast primary
Breast vHMEC
Penis foreskin keratinocyte primary
Placenta amnion
Placenta chorion
Psoas muscle
Right ventricle
Breast myoepithelial
Ovary
Right atrium
Left Ventricle
Lung
Duodenum smooth muscle
Penis foreskin fibroblast primary
Fetal lung
Fetal stomach
Rectal smooth muscle
Colon smooth muscle
Stomach smooth muscle
Fetal leg muscle
Fetal trunk muscle
Breast luminal epithelial
Penis foreskin melanocyte primary
Mid frontal lobe
Fetal brain
Inferior temporal lobe
Anterior caudate
Cingulate gyrus
Hippocampus middle
Angular gyrus
Substantia nigra
CD15 primary
CD14 primary
CD19 primary (BI)
CD19 primary (UW)
CD8 naive primary (BI)
CD8 naive primary (UCSF-UBC)
CD3 primary (BI)
CD4+ CD25- CD45RA+ naive primary
CD4+ CD25- CD45R0+ memory primary
CD4 memory primary
CD4 naive primary
CD8 memory primary
CD4+ CD25- IL17- PMA Ionomycin stim MACS Th sprimary
CD4+ CD25- Th primary
CD4+ CD25+ CD127- Treg primary
CD4+ CD25int CD127+ Tmem primary
CD4+ CD25- IL17+ PMA Ionomycin stim Th17 primary
CD3 primary (UW)
CD56 primary
CD34 primary
Mobilized CD34 primary
Peripheralblood mononuclear primary
Thymus
Fetal thymus
Vitamin D
UKBiobank Age at Menopause
Lupus
Primary Biliary Cirrhosis
UKBiobank Asthma
Inflammatory Bowel Disease
Ulcerative Colitis
Crohns Disease
Celiac Disease
UKBiobank Eczema
Multiple Sclerosis
Rheumatoid Arthritis
Anorexia
Bipolar Disorder
Subject Well Being
Depressive Symptoms
UKBiobank Age at Menarche
UKBiobank BMI
Ever smoked
Neuroticism
Schizophrenia
Autism
Age-related Macular Degeneration
Type2 Diabetes
UKBiobank Waist Hip Ratio
UKBiobank Diastolic Blood Pressure
UKBiobank Systolic Blood Pressure
UKBiobank FEV1FVC ratio
UKBiobank Heel T-Score
UKBiobank Forced Vital Capacity
UKBiobank Height
Alzheimer
Triglycerides
High-density Lipoprotein
Low-density Lipoprotein
Fasting Glucose
UKBiobank Hypertension
Coronary Artery Disease

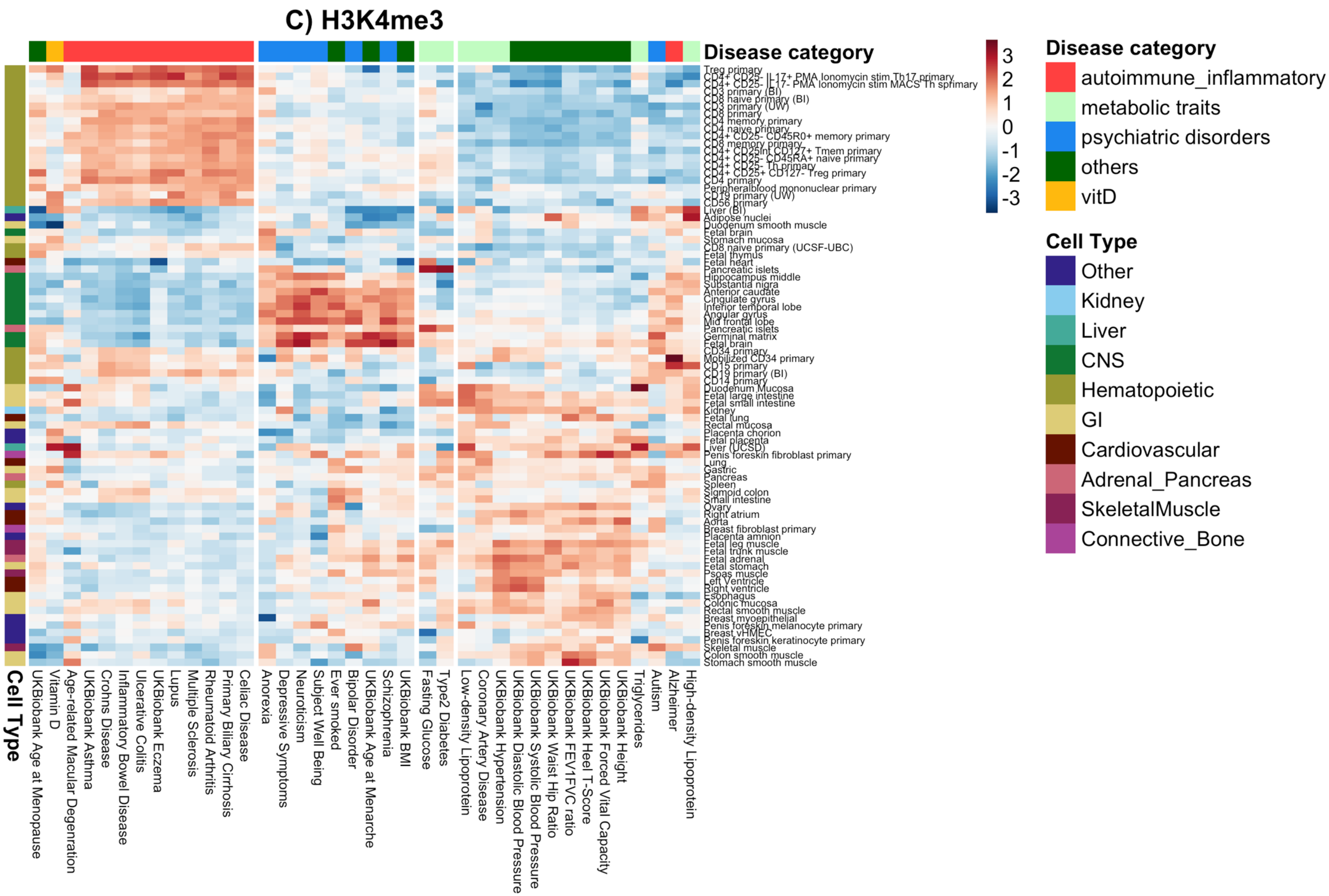
C) H3K4me3
Disease category
Treg primary
CD4+ CD25- IL17+ PMA Ionomycin stim Th17 primary
CD4+ CD25- IL17- PMA Ionomycin stim MACS Th primary
CD3 primary (BI)
CD8 naive primary (BI)
CD3 primary (UW)
CD8 primary
CD4 memory primary
CD4 naive primary
CD4+ CD25- CD45R0+ memory primary
CD8 memory primary
CD4+ CD25int CD127+ Tmem primary
CD4+ CD25- CD45RA+ naive primary
CD4+ CD25- Th primary
CD4+ CD25+ CD127- Treg primary
CD4 primary
Peripheralblood mononuclear primary
CD19 primary (UW)
CD56 primary
Liver (BI)
Adipose nuclei
Duodenum smooth muscle
Fetal brain
Stomach mucosa
CD8 naive primary (UCSF-UBC)
Fetal thymus
Fetal heart
Pancreatic islets
Hippocampus middle
Substantia nigra
Anterior caudate
Cingulate gyrus
Inferior temporal lobe
Angular gyrus
Mid frontal lobe
Pancreatic islets
Germinal matrix
Fetal brain
CD34 primary
Mobilized CD34 primary
CD15 primary
CD19 primary (BI)
CD14 primary
Duodenum Mucosa
Fetal large intestine
Fetal small intestine
Kidney
Fetal lung
Rectal mucosa
Placenta chorion
Fetal placenta
Liver (UCSD)
Penis foreskin fibroblast primary
Lung
Gastric
Pancreas
Spleen
Sigmoid colon
Small intestine
Ovary
Right atrium
Aorta
Breast fibroblast primary
Placenta amnion
Fetal leg muscle
Fetal trunk muscle
Fetal adrenal
Fetal stomach
Psoas muscle
Left Ventricle
Right ventricle
Esophagus
Colonic mucosa
Rectal smooth muscle
Breast myoepithelial
Penis foreskin melanocyte primary
Breast vHMEC
Penis foreskin keratinocyte primary
Skeletal muscle
Colon smooth muscle
Stomach smooth muscle
Cell Type
UKBiobank Age at Menopause
Vitamin D
Age-related Macular Degeneration
UKBiobank Asthma
Crohns Disease
Inflammatory Bowel Disease
Ulcerative Colitis
UKBiobank Eczema
Lupus
Multiple Sclerosis
Rheumatoid Arthritis
Primary Biliary Cirrhosis
Celiac Disease
Anorexia
Depressive Symptoms
Neuroticism
Subject Well Being
Ever smoked
Bipolar Disorder
UKBiobank Age at Menarche
Schizophrenia
UKBiobank BMI
Fasting Glucose
Type2 Diabetes
Low-density Lipoprotein
Coronary Artery Disease
UKBiobank Hypertension
UKBiobank Diastolic Blood Pressure
UKBiobank Systolic Blood Pressure
UKBiobank Waist Hip Ratio
UKBiobank FEV1FVC ratio
UKBiobank Heel T-Score
UKBiobank Forced Vital Capacity
UKBiobank Height
Triglycerides
Autism
Alzheimer
High-density Lipoprotein
3
2
1
0
-1
-2
-3
Disease category
autoimmune_inflammatory
metabolic traits
psychiatric disorders
others
vitD
Cell Type
Other
Kidney
Liver
CNS
Hematopoietic
GI
Cardiovascular
Adrenal_Pancreas
SkeletalMuscle
Connective_Bone

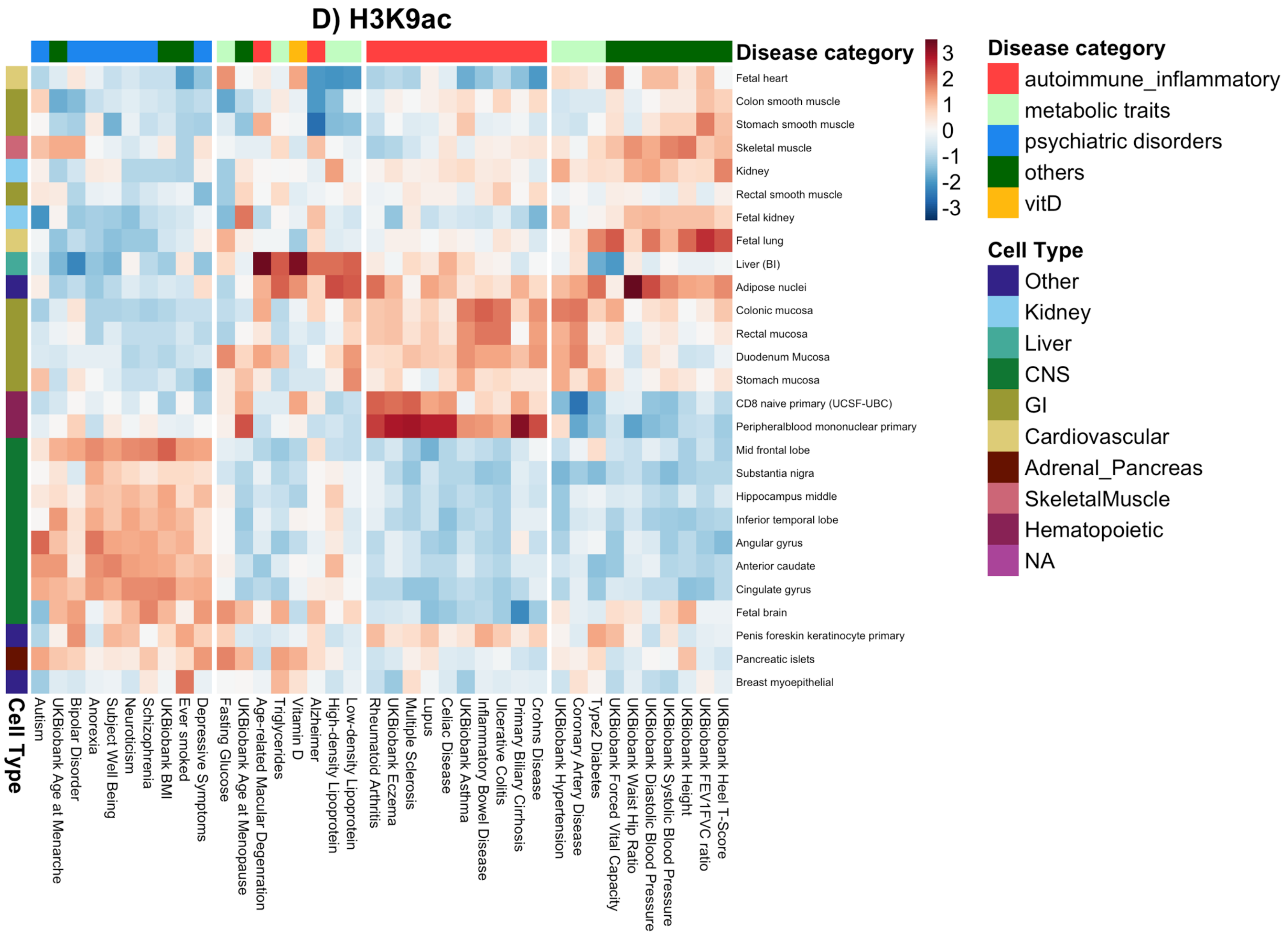

Supplementary Figure 5. Heat-maps showing bi-clustering of traits and cell-types over four histone marks.
We performed 220 cell-type-specific annotation analysis in each of the 37 traits, and compared these enrichment results to the enrichment results of 25-hydroxyvitamin D. Each checker reflects the beta coefficient Z-score, scaled by traits. Red indicates enrichment, blue indicates depletion. Deeper color represents stronger magnitude of significance. The category of each disease/trait is color coded to the top, and the category of cell types is color coded to the left. A) H3K27ac, B) H3K4me1, C) H3K4me3 and D) H3K9ac. GI: gastrointestinal cell types; CNS: central nervous system cell types.

Supplementary Note2

Acknowledgements

1958 British Birth Cohort (1958BC) The project was funded by the UK Medical Research Council (MRC) (project grant G0601653), and 25-hydroxyvitamin D assays by the BUPA foundation. EH is funded by a UK Department of Health Public Health Career Scientist Award. Use of DNA from this cohort was funded by MRC grant G0000934 and Wellcome Trust grant 068545/Z/02. This research used resources provided by the Type 1 Diabetes Genetics Consortium, a collaborative clinical study sponsored by the National Institute of Diabetes and Digestive and Kidney Diseases (NIDDK), National Institute of Allergy and Infectious Diseases, National Human Genome Research Institute, National Institute of Child Health and Human Development, and Juvenile Diabetes Research Foundation International (JDRF) and supported by U01 DK062418. This study makes use of data generated by the Wellcome Trust Case-Control Consortium. A full list of investigators who contributed to generation of the data is available from the Wellcome Trust Case-Control Consortium website. Funding for the project was provided by the Wellcome Trust under award 076113. Great Ormond Street Hospital/University College London, Institute of Child Health receives a proportion of funding from the Department of Health's NIHR (Biomedical Research Centres funding). MMcC is a Wellcome Trust Senior Investigator supported by Wellcome Trust awards 098381 and 090532.

Cardiovascular Health Study (CHS) Research for this cohort that was reported in this article was supported by contract numbers N01-HC-85079 to N01-HC-85086, N01-HC-35129, N01 HC-15103, N01-HC-55222, N01-HC-75150, N01-HC-45133, grant numbers U01 HL080295, R01 HL084443, R01 HL087652, and R01 AG027002 from NHLBI, with additional contribution from the National Institute of Neurological Disorders and Stroke. A full list of principal investigators and institutions is available at the Cardiovascular Health Study website. DNA handling and genotyping was supported in part by National Center for Research Resources grant M01RR00069 to the Cedars-Sinai General Clinical Research Center Genotyping core and NIDDK grant DK063491 to the Southern California Diabetes Endocrinology Research Center.

Framingham Heart Study (FHS) Framingham Heart Study of the National Heart, Lung and Blood Institute (NHLBI) of the US National Institutes of Health (NIH) and Boston University School of Medicine is supported by the NIH/NHLBI contracts N01-HC-25195 and HHSN268201500001I. The present study received support from the American Heart Association, the US Department of Agriculture, Agricultural Research Service (under Cooperative Agreement No 58-1950-7-707), the National Institute of Aging (AG14759), the US National Institute for Arthritis, Musculoskeletal and Skin Diseases (R01AR057118), and the US National Institute for Arthritis, Musculoskeletal and Skin Diseases and National Institute on Aging (R01 AR 41398). DK was supported by a grant from the National Institute of Arthritis, Musculoskeletal, and Skin Diseases and the National Institute on Aging (R01 AR/AG 41398), and also by FP7-PEOPLE-2012-Marie Curie Career Integration Grants (CIG)). The analyses reflect intellectual input and resource development from the Framingham Heart Study investigators participating in the SNP Health Association Resource project. This work was partly supported by a contract with Affymetrix Inc for genotyping services (Contract No N02-HL-6-4278). A portion of this research used the Linux Cluster for Genetic Analysis, which is funded by the Robert Dawson Evans Endowment of the Department of Medicine at Boston University School of Medicine and Boston Medical Center.

Gothenberg Osteoporosis and Obesity Determinants (GOOD) Study Financial support was received from the Swedish Research Council, the Swedish Foundation for Strategic Research, The ALF/LUA research grant in Gothenburg, the Lundberg Foundation, the Emil and Vera Cornell Foundation, the Torsten and Ragnar S.derberg's Foundation, Petrus and Augusta Hedlunds Foundation, the V.stra G.taland Foundation, the G.teborg Medical Society, and the Sahlgrenska Center for Cardiovascular and Metabolic Research (CMR, no A305:188), which is supported by the Swedish Strategic Foundation.

Health, Aging and Body Composition Study (Health ABC) This research was supported by the Intramural Research Program of the National Institutes of Health, National Institute on Aging and National Institute on Aging contracts N01-AG-6-2101, N01-AG-6-2103, N01-AG-6-2106, NIA

grant R01-AG028050, and NINR grant R01-NR012459. Assessment of 25-hydroxyvitamin D concentrations was funded by a National Institute on Aging grant, R01-AG029364. The genome-wide association study was funded by a National Institute on Aging grant, R01-AG032098, and genotyping services were provided by CIDR. CIDR is fully funded through a federal contract from NIH to The Johns Hopkins University (contract number HHSN268200782096C).

Study of Indiana Women This work was supported by NIH grants P01 AG-18397, M01 RR-00750, and R01 AG-041517. Genotyping services were provided by CIDR. CIDR is fully funded through a federal contract from NIH to The Johns Hopkins University, contract number HHSN268200782096C. This research was supported in part by the Intramural Research Program of the NIH, National Library of Medicine.

North Finland Birth Cohort 1966 (NFBC) We thank the late Professor Paula Rantakallio (launch of NFBC1966), Ms Outi Tornwall and Ms Minttu Jussila (DNA biobanking). The authors would like to acknowledge the contribution of the late Academician of Science Leena Peltonen. The NFBC resource has been supported by grants from the Academy of Finland (project grants 104781, 120315, 129269, 1114194, 24300796, Center of Excellence in Complex Disease Genetics and SALVE), University Hospital Oulu, Biocenter, University of Oulu, Finland (75617), NHLBI grant 5R01HL087679-02 (1RL1MH083268-01), NIH/NIMH (5R01MH63706:02), ENGAGE project and grant agreement HEALTH-F4-2007-201413, EU FP7 EurHEALTHAgeing -277849, the Medical Research Council, UK (G0500539, G0600705, G1002319, PrevMetSyn/SALVE) and the MRC, Centenary Early Career Award. H2020 DynaHEALTH (European Union's Horizon 2020 research and innovation programme under grant agreement No 633595); Exposomic, Genomic and Epigenomic Approach to Prediction of Metabolic and Cardiorespiratory function and Ill-Health (EGEA), Academy of Finland, Grant No 285547; ALEC Study (funded by the European Union's Horizon 2020 Research and Innovation programme under grant agreement No. 633212); H2020 / Marie Skłodowska-Curie Actions, CAPICE (Marie Curie Grant agreement Number 721567); National Public Health Institute, Biomedicum Helsinki, Finland.

The Amish Family Osteoporosis Study (OOA) was funded by a grant from the National Institute of Arthritis, Musculoskeletal and Skin Diseases (R01 AR46838).

The Rotterdam Study The generation and management of GWAS genotype data for the Rotterdam Study (RS I, RS II, RS III) was executed by the Human Genotyping Facility of the Genetic Laboratory of the Department of Internal Medicine, Erasmus MC, Rotterdam, The Netherlands. The GWAS datasets are supported by the Netherlands Organization of Scientific Research NWO Investments (nr. 175.010.2005.011, 911-03-012), the Genetic Laboratory of the Department of Internal Medicine, Erasmus MC, the Research Institute for Diseases in the Elderly (014-93-015; RIDE2), the Netherlands Genomics Initiative (NGI)/Netherlands Organisation for Scientific Research (NWO) Netherlands Consortium for Healthy Aging (NCHA), project nr. 050-060-810. We thank Pascal Arp, Mila Jhamai, Marijn Verkerk, Lizbeth Herrera and Marjolein Peters, MSc, and Carolina Medina-Gomez, MSc, for their help in creating the GWAS database, and Karol Estrada, PhD, Yurii Aulchenko, PhD, and Carolina Medina-Gomez, PhD, for the creation and analysis of imputed data. We would like to thank Dr. Karol Estrada, Dr. Fernando Rivadeneira, Dr. Tobias A. Knoch, Marijn Verkerk, Anis Abuseiris, Dr. Linda Boer and Rob de Graaf (Erasmus MC Rotterdam, The Netherlands), for their help in creating and maintaining GRIMP. The Rotterdam Study is funded by Erasmus Medical Center and Erasmus University, Rotterdam, Netherlands Organization for the Health Research and Development (ZonMw), the Research Institute for Diseases in the Elderly (RIDE), the Ministry of Education, Culture and Science, the Ministry for Health, Welfare and Sports, the European Commission (DG XII), and the Municipality of Rotterdam. The authors are very grateful to the study participants, the staff from the Rotterdam Study (particularly L. Buist and J.H. van den Boogert) and the participating general practitioners and pharmacists. DSM Nutritional Products AG, Kaiseraugst, Switzerland provided funding for the assessment of vitamin D.

TwinsUK was funded by the Wellcome Trust, Arthritis Research Campaign, European Community's Seventh Framework Programme (FP7/2007-2013)/grant agreement HEALTH-F2-2008-201865-GEFOS and Seventh Framework Programme grant 200800 Treat OA /(FP7/2007-2013), ENGAGE

project grant agreement HEALTH-F4-2007-201413, and the FP-5 GenomEUtwin Project (QLG2-CT-2002-01254). The study also receives support from the UK Department of Health via the National Institute for Health Research (NIHR) comprehensive Biomedical Research Centre award to Guy's & St Thomas' NHS Foundation Trust in partnership with King's College London. TDS is an NIHR senior investigator. The project also received support from a Biotechnology and Biological Sciences Research Council project grant. (G20234). The authors acknowledge the funding and support of the National Eye Institute (NEI) via an NIH/Center for Inherited Disease Research (CIDR) genotyping project. We thank the staff from the Genotyping Facilities at the Wellcome Trust Sanger Institute for sample preparation, quality control, and genotyping; Le Centre National de Gnotypage, France, for genotyping; Duke University, NC, USA, for genotyping; and the Finnish Institute of Molecular Medicine, Finnish Genome Center, University of Helsinki. Genotyping was also done by CIDR as part of an NEI/NIH project grant.

Alpha-Tocopherol, Beta-Carotene Cancer Prevention Study (ATBC) is supported by the Intramural Research Program of the U.S. National Cancer Institute, National Institutes of Health, and by U.S. Public Health Service contract HHSN261201500005C from the National Cancer Institute, Department of Health and Human Services.

The Prostate, Lung, Colorectal and Ovarian Cancer Screening Trial (PLCO) is supported by the Intramural Research Program of the Division of Cancer Epidemiology and Genetics and by contracts from the Division of Cancer Prevention, National Cancer Institute (NCI), US National Institutes of Health (NIH), Department of Health and Human Services (DHHS).

Atherosclerosis Risk in Communities (ARIC) Study ARIC's contribution to this project was supported by NHLBI R01 HL103706 and contracts (HHSN268201100005C, HHSN268201100006C, HHSN268201100007C,HHSN268201100008C, HHSN268201100009C, HHSN268201100010C, HHSN268201100011C, and HHSN268201100012C), R01HL087641, R01HL59367 and R01HL086694; Office of Dietary Supplements grant number R01 HL103706-S1, National Human Genome Research Institute contract U01HG004402; and National Institutes of Health contract HHSN268200625226C. Infrastructure was partly supported by Grant Number UL1RR025005, a component of the National Institutes of Health and NIH Roadmap for Medical Research. The authors thank the staff and participants of the ARIC study for their important contributions.

B-vitamins of the Prevention of Osteoporotic Fractures (B-PROOF) is supported and funded by The Netherlands Organization for Health Research and Development (ZonMw, Grant 6130.0031), the Hague; unrestricted grant from NZO (Dutch Dairy Association), Zoetermeer; MCO Health, Almere; NCHA (Netherlands Consortium Healthy Ageing) Leiden/ Rotterdam; Ministry of Economic Affairs, Agriculture and Innovation (project KB-15-004-003), the Hague; Wageningen University, Wageningen; VU University Medical Center, Amsterdam; Erasmus MC, Rotterdam. The authors gratefully thank all study participants, and all co-workers who helped to succeed this trial, especially P.H. in 't Veld, M. Hillen-Tijdink, A. Nicolaas-Merkus, N. Pliester, S. Oliai Araghi, and S. Smits. They also thank Professor Dr. H.A.P. Pols for obtaining funding.

Leiden Longevity Study (LLS) has received funding from the European Union's Seventh Framework Programme (FP7/2007-2011) under grant agreement number 259679 (IDEAL). This study was financially supported by the Innovation-Oriented Research Program on Genomics (SenterNovem IGE05007), the Centre for Medical Systems Biology and the Netherlands Consortium for Healthy Ageing (grant 050-060-810), all in the framework of the Netherlands Genomics Initiative, Netherlands Organization for Scientific Research (NWO), by Unilever Colworth and by BBMRI-NL, a Research Infrastructure financed by the Dutch government (NWO 184.021.007).

Ludwigshafen Risk and Cardiovascular Health Study (LURIC) is supported by the 7th Framework Program (integrated project AtheroRemo, grant agreement number 201668 and RiskyCAD, grant agreement number 305739) of the European Union. The work of M.E.K. was supported by the German Federal Ministry of Education and Research (grant number 01EA1411A).

Multi-Ethnic Study of Atherosclerosis (MESA) MESA and the MESA SHARe project are conducted and supported by the National Heart, Lung, and Blood Institute (NHLBI) in collaboration with MESA investigators. Support for MESA is provided by contracts HHSN268201500003I, N01-HC-

95159, N01-HC-95160, N01-HC-95161, N01-HC-95162, N01-HC-95163, N01-HC-95164, N01-HC-95165, N01-HC-95166, N01-HC-95167, N01-HC-95168, N01-HC-95169, UL1-TR-000040, UL1-TR-001079, UL1-TR-001420, UL1-TR-001881, and DK063491. Support for the Mineral Metabolite dataset was provided by grant HL096875.

The Nurses' Health Study (NHS) is supported by NIH grants P01CA087969 and 5U01HG004399-2. XJ is supported by VetenskarpsRadet International Postdoc Grant.

The Health Professionals Follow-up Study (HPFS) is supported by NIH grant P01CA055075. The American Cancer Society (ACS) study is supported by U01 CA098710. K.C.S. is supported by NIH Kirschstein-NRSA T32 ES016645-01. The genome-wide scans have been supported by the NCI, NIH, under contract N01-CO-12400.

The Orkney Complex Disease Study (ORCADES) was supported by the Chief Scientist Office of the Scottish Government (CZB/4/276, CZB/4/710), the Royal Society, the MRC Human Genetics Unit quinquennial programme "QTL in Health and Disease", Arthritis Research UK and the European Union framework program 6 EUROSPAN project (contract no. LSHG-CT-2006-018947). DNA extractions were performed at the Wellcome Trust Clinical Research Facility in Edinburgh. We would like to acknowledge the invaluable contributions of the research nurses in Orkney, the administrative team in Edinburgh and the people of Orkney.

Prospective Study of Pravastatin in the Elderly at Risk (PROSPER) was supported by an investigator initiated grant obtained from Bristol-Myers Squibb. Professor Dr. J. W. Jukema is an Established Clinical Investigator of the Netherlands Heart Foundation (grant 2001 D 032). Support for genotyping was provided by the seventh framework program of the European commission (grant 223004) and by the Netherlands Genomics Initiative (Netherlands Consortium for Healthy Aging grant 050-060-810).

The Scottish Colorectal Cancer Study (SOCCS) The work was funded by grants from Cancer Research UK (C348/A3758, C348/A8896, C348/A18927); Scottish Government Chief Scientist Office (K/OPR/2/2/D333, CZB/4/94, CZB/4/449); Medical Research Council (G0000657-53203, MR/K018647/1); Centre Grant from CORE as part of the Digestive Cancer Campaign (http://www.corecharity.org.uk). We thank all the participants and all the recruitment teams and collaborators who make such studies possible. We acknowledge the excellent technical support from Marion Walker. We are grateful to Ruth Wilson, Donna Markie, and all those who continue to contribute to recruitment, data collection, and data curation for the Study of Colorectal Cancer in Scotland studies. We acknowledge the expert support on sample preparation from the Genetics Core of the Edinburgh Wellcome Trust Clinical Research Facility.

The Young Finns Study (YFS) has been financially supported by the Academy of Finland: grants 286284, 134309 (Eye), 126925, 121584, 124282, 129378 (Salve), 117787 (Gendi), and 41071 (Skidi); the Social Insurance Institution of Finland; Competitive State Research Financing of the Expert Responsibility area of Kuopio, Tampere and Turku University Hospitals (grant X51001); Juho Vainio Foundation; Paavo Nurmi Foundation; Finnish Foundation for Cardiovascular Research; Finnish Cultural Foundation; Tampere Tuberculosis Foundation; Emil Aaltonen Foundation; Yrjö Jahnsson Foundation; Signe and Ane Gyllenberg Foundation; and Diabetes Research Foundation of Finnish Diabetes Association. The expert technical assistance in the statistical analyses by Irina Lisinen is gratefully acknowledged.

Helsinki Birth Cohort Study (HBCS) We thank all study participants as well as everybody involved in the Helsinki Birth Cohort Study (HBCS). HBCS was financially supported by Academy of Finland (grant no. 129369, 129907, 135072, 129255, and 126775), Finnish Foundation for Diabetes Research, Novo Nordisk Foundation, Signe and Ane Gyllenberg Foundation, Samfundet Folkhälsan, Finska Läkaresällskapet, Liv och Hälsa, European Commission within the 7th Framework Programme (DORIAN, grant agreement no. 278603), and European Union Horizon 2020 programme (DYNAHEALTH grant no. 633595).

The EPIC-InterAct is supported by EU-FP6 EU FP6 programme (LSHM_CT_2006_037197); Medical Research Council Epidemiology Unit (MC_UU_12015/1); Cambridge Initiative (RG71466, SJAH/004); NIHR Biomedical Research Centre Cambridge: Nutrition, Diet, and Lifestyle Research Theme (IS-BRC-1215-20014).

The EPIC-Norfolk study received grants from the Medical Research Council (G9502233) and Cancer Research UK (SP2024-0201 and SP2024-0204).

CHD case ascertainment and validation, genotyping, and clinical chemistry assays in **EPIC-CVD** were supported by grants awarded to the University of Cambridge from the EU Framework Programme 7 (HEALTH-F2-2012-279233), the UK Medical Research Council (G0800270) and British Heart Foundation (SP/09/002), and the European Research Council (268834).

The authors would like to thank Timothy R Gailius for his excellent assistance with information collection.

Supplementary References

1. Wang, T. J. *et al*. Common genetic determinants of vitamin D insufficiency: a genome-wide association study. *Lancet Lond. Engl.* **376,** 180–188 (2010).
2. Psaty, B. M. *et al*. Cohorts for Heart and Aging Research in Genomic Epidemiology (CHARGE) Consortium: Design of prospective meta-analyses of genome-wide association studies from 5 cohorts. *Circ. Cardiovasc. Genet.* **2,** 73–80 (2009).
3. The alpha-tocopherol, beta-carotene lung cancer prevention study: design, methods, participant characteristics, and compliance. The ATBC Cancer Prevention Study Group. *Ann. Epidemiol.* **4,** 1–10 (1994).
4. Ahn, J. *et al*. Genome-wide association study of circulating vitamin D levels. *Hum. Mol. Genet.* **19,** 2739–2745 (2010).
5. The Atherosclerosis Risk in Communities (ARIC) Study: design and objectives. The ARIC investigators. *Am. J. Epidemiol.* **129,** 687–702 (1989).
6. Blankenberg, S. *et al*. Glutathione peroxidase 1 activity and cardiovascular events in patients with coronary artery disease. *N. Engl. J. Med.* **349,** 1605–1613 (2003).
7. van Wijngaarden, J. P. *et al*. Rationale and design of the B-PROOF study, a randomized controlled trial on the effect of supplemental intake of vitamin B12 and folic acid on fracture incidence. *BMC Geriatr.* **11,** 80 (2011).
8. Epidemiology of Diabetes Interventions and Complications (EDIC). Design, implementation, and preliminary results of a long-term follow-up of the Diabetes Control and Complications Trial cohort. *Diabetes Care* **22,** 99–111 (1999).
9. Paterson, A. D. *et al*. A genome-wide association study identifies a novel major locus for glycemic control in type 1 diabetes, as measured by both A1C and glucose. *Diabetes* **59,** 539–549 (2010).
10. Kristiansson, K. *et al*. Genome-wide screen for metabolic syndrome susceptibility Loci reveals strong lipid gene contribution but no evidence for common genetic basis for clustering of metabolic syndrome traits. *Circ. Cardiovasc. Genet.* **5,** 242–249 (2012).
11. Eriksson, J. G. Early growth and coronary heart disease and type 2 diabetes: findings from the Helsinki Birth Cohort Study (HBCS). *Am. J. Clin. Nutr.* **94,** 1799S–1802S (2011).
12. Rimm, E. B. *et al*. Prospective study of alcohol consumption and risk of coronary disease in men. *Lancet Lond. Engl.* **338,** 464–468 (1991).
13. Ferrucci, L. *et al*. Subsystems contributing to the decline in ability to walk: bridging the gap between epidemiology and geriatric practice in the InCHIANTI study. *J. Am. Geriatr. Soc.* **48,** 1618–1625 (2000).
14. Tanaka, T. *et al*. Genome-Wide Association Study of Plasma Polyunsaturated Fatty Acids in the InCHIANTI Study. *PLOS Genet.* **5,** e1000338 (2009).
15. Holle, R., Happich, M., Löwel, H., Wichmann, H. E. & MONICA/KORA Study Group. KORA--a research platform for population based health research. *Gesundheitswesen Bundesverb. Arzte Offentlichen Gesundheitsdienstes Ger.* **67 Suppl 1,** S19-25 (2005).

16. Granada, M. *et al.* A Genome Wide Association Study of Plasma Total IgE Concentration in the Framingham Heart Study. *J. Allergy Clin. Immunol.* **129,** 840–845.e21 (2012).
17. Schoenmaker, M. *et al.* Evidence of genetic enrichment for exceptional survival using a family approach: the Leiden Longevity Study. *Eur. J. Hum. Genet. EJHG* **14,** 79–84 (2006).
18. Deelen, J. *et al.* Genome-wide association study identifies a single major locus contributing to survival into old age; the APOE locus revisited. *Aging Cell* **10,** 686–698 (2011).
19. Winkelmann, B. R. *et al.* Rationale and design of the LURIC study--a resource for functional genomics, pharmacogenomics and long-term prognosis of cardiovascular disease. *Pharmacogenomics* **2,** S1-73 (2001).
20. Kleber, M. E. *et al.* Genome-wide association study identifies 3 genomic loci significantly associated with serum levels of homoarginine: the AtheroRemo Consortium. *Circ. Cardiovasc. Genet.* **6,** 505–513 (2013).
21. Rasmussen-Torvik, L. J. *et al.* Fasting glucose GWAS candidate region analysis across ethnic groups in the Multiethnic Study of Atherosclerosis (MESA). *Genet. Epidemiol.* **36,** 384–391 (2012).
22. Galesloot, T. E. *et al.* Cohort Profile: The Nijmegen Biomedical Study (NBS). *Int. J. Epidemiol.* (2017). doi:10.1093/ije/dyw268
23. Colditz, G. A. & Hankinson, S. E. The Nurses' Health Study: lifestyle and health among women. *Nat. Rev. Cancer* **5,** 388–396 (2005).
24. Tworoger, S. S., Sluss, P. & Hankinson, S. E. Association between plasma prolactin concentrations and risk of breast cancer among predominately premenopausal women. *Cancer Res.* **66,** 2476–2482 (2006).
25. Hunter, D. J. *et al.* A genome-wide association study identifies alleles in FGFR2 associated with risk of sporadic postmenopausal breast cancer. *Nat. Genet.* **39,** 870–874 (2007).
26. McQuillan, R. *et al.* Runs of homozygosity in European populations. *Am. J. Hum. Genet.* **83,** 359–372 (2008).
27. Kramer, B. S., Gohagan, J., Prorok, P. C. & Smart, C. A National Cancer Institute sponsored screening trial for prostatic, lung, colorectal, and ovarian cancers. *Cancer* **71,** 589–593 (1993).
28. Yeager, M. *et al.* Genome-wide association study of prostate cancer identifies a second risk locus at 8q24. *Nat. Genet.* **39,** 645–649 (2007).
29. Shepherd, J. *et al.* The design of a prospective study of Pravastatin in the Elderly at Risk (PROSPER). PROSPER Study Group. PROspective Study of Pravastatin in the Elderly at Risk. *Am. J. Cardiol.* **84,** 1192–1197 (1999).
30. Trompet, S. *et al.* Replication of LDL GWAs hits in PROSPER/PHASE as validation for future (pharmaco)genetic analyses. *BMC Med. Genet.* **12,** 131 (2011).
31. Hensel, E. *et al.* Study of Health in Pomerania (SHIP): a health survey in an East German region. Objectives and design of the oral health section. *Quintessence Int. Berl. Ger. 1985* **34,** 370–378 (2003).
32. Völzke, H. [Study of Health in Pomerania (SHIP). Concept, design and selected results]. *Bundesgesundheitsblatt Gesundheitsforschung Gesundheitsschutz* **55,** 790–794 (2012).
33. Völzke, H. *et al.* Cohort profile: the study of health in Pomerania. *Int. J. Epidemiol.* **40,** 294–307 (2011).
34. Tenesa, A. *et al.* Genome-wide association scan identifies a colorectal cancer susceptibility locus on 11q23 and replicates risk loci at 8q24 and 18q21. *Nat. Genet.* **40,** 631–637 (2008).
35. Tomlinson, I. P. M. *et al.* Multiple Common Susceptibility Variants near BMP Pathway Loci GREM1, BMP4, and BMP2 Explain Part of the Missing Heritability of Colorectal Cancer. *PLoS Genet.* **7,** (2011).
36. Raitakari, O. T. *et al.* Cohort profile: the cardiovascular risk in Young Finns Study. *Int. J. Epidemiol.* **37,** 1220–1226 (2008).
37. Smith, E. N. *et al.* Longitudinal genome-wide association of cardiovascular disease risk factors in the Bogalusa heart study. *PLoS Genet.* **6,** e1001094 (2010).

38. Lind, L., Fors, N., Hall, J., Marttala, K. & Stenborg, A. A comparison of three different methods to evaluate endothelium-dependent vasodilation in the elderly: the Prospective Investigation of the Vasculature in Uppsala Seniors (PIVUS) study. *Arterioscler. Thromb. Vasc. Biol.* **25,** 2368–2375 (2005).
39. Fan, J. B. *et al.* Highly parallel SNP genotyping. *Cold Spring Harb. Symp. Quant. Biol.* **68,** 69–78 (2003).
40. Ho, J. E. *et al.* Clinical and genetic correlates of growth differentiation factor 15 in the community. *Clin. Chem.* **58,** 1582–1591 (2012).
41. Rönnemaa, E. *et al.* Impaired insulin secretion increases the risk of Alzheimer disease. *Neurology* **71,** 1065–1071 (2008).
42. den Hoed, M. *et al.* GWAS-identified loci for coronary heart disease are associated with intima-media thickness and plaque presence at the carotid artery bulb. *Atherosclerosis* **239,** 304–310 (2015).
43. InterAct Consortium *et al.* Design and cohort description of the InterAct Project: an examination of the interaction of genetic and lifestyle factors on the incidence of type 2 diabetes in the EPIC Study. *Diabetologia* **54,** 2272–2282 (2011).
44. Danesh, J. *et al.* EPIC-Heart: the cardiovascular component of a prospective study of nutritional, lifestyle and biological factors in 520,000 middle-aged participants from 10 European countries. *Eur. J. Epidemiol.* **22,** 129–141 (2007).
45. Day, N. *et al.* EPIC-Norfolk: study design and characteristics of the cohort. European Prospective Investigation of Cancer. *Br. J. Cancer* **80 Suppl 1,** 95–103 (1999).
46. Khaw, K.-T., Luben, R. & Wareham, N. Serum 25-hydroxyvitamin D, mortality, and incident cardiovascular disease, respiratory disease, cancers, and fractures: a 13-y prospective population study. *Am. J. Clin. Nutr.* **100,** 1361–1370 (2014).
