# Supplementary material for: Genome-wide association study in 79,366 European-ancestry individuals informs the genetic architecture of 25-hydroxyvitamin D levels
Source: Nat Commun. 2018 Jan 17;9:260. doi: 10.1038/s41467-017-02662-2 (PMC5772647; doi:10.1038/s41467-017-02662-2)
Supplement: Supplementary file 3 — Description of Additional Supplementary Files [file 41467_2017_2662_MOESM3_ESM.pdf]

Description of Supplementary Files

File Name: Supplementary Data 1
Description: Enrichment of 220 cell-type-specific annotations.
