# Supplementary material for: Genome-wide association study in 79,366 European-ancestry individuals informs the genetic architecture of 25-hydroxyvitamin D levels
Source: Nat Commun. 2018 Jan 17;9:260. doi: 10.1038/s41467-017-02662-2 (PMC5772647; doi:10.1038/s41467-017-02662-2)
Supplement: Supplementary file 4 — Supplementary data 1 [file 41467_2017_2662_MOESM4_ESM.docx]

Genome-wide Association Study in 79,366 European-ancestry Individuals Informs the Genetic Architecture of 25-Hydroxyvitamin D Levels

Xia Jiang *et al.*

| **Supplementary Data 1. Enrichment of 220 cell-type-specific annotations.** | | | | | | |
| --- | --- | --- | --- | --- | --- | --- |
| **Cell Type** | **Histone Mark** | **Proportion of SNPs (%)** | **Proportion of h2** | **Enrichment (standard error)** | **Enrichment P-values** | **category** |
| CD19 primary (UW) | H3K4me1 | 4.10 | 32.85 | 8.01(2.07) | **0.00067** | **Hematopoietic/Immune** |
| CD19 | H3K27ac | 3.66 | 29.66 | 8.11(2.2) | **0.00071** | **Hematopoietic/Immune** |
| CD19 primary (BI) | H3K4me1 | 4.09 | 32.29 | 7.9(2.24) | **0.00194** | **Hematopoietic/Immune** |
| CD20 | H3K27ac | 2.87 | 18.41 | 6.41(1.93) | **0.0027** | **Hematopoietic/Immune** |
| CD3 primary | H3K27ac | 3.19 | 24.89 | 7.79(2.67) | **0.01116** | **Hematopoietic/Immune** |
| Liver (BI) | H3K4me1 | 5.50 | 32.95 | 5.99(1.9) | **0.01256** | **Liver** |
| Liver | H3K27ac | 2.61 | 21.91 | 8.38(2.96) | **0.01405** | **Liver** |
| CD4+ CD25- IL17+ PMA Ionomycin stim Th17 primary | H3K4me1 | 3.95 | 22.31 | 5.66(2.02) | **0.01725** | **Hematopoietic/Immune** |
| CD15 primary | H3K4me1 | 3.64 | 21.55 | 5.92(2.16) | **0.01973** | **Hematopoietic/Immune** |
| Right atrium | H3K4me1 | 0.92 | 15.95 | 17.3(7.58) | **0.02029** | **Cardiovascular** |
| CD56 primary | H3K4me1 | 3.49 | 21.61 | 6.19(2.31) | **0.02182** | **Hematopoietic/Immune** |
| Mobilized CD34 primary | H3K4me1 | 6.92 | 31.6 | 4.57(1.69) | **0.02637** | **Hematopoietic/Immune** |
| Penis foreskin keratinocyte primary | H3K4me1 | 6.09 | 37.24 | 6.12(2.41) | **0.02922** | **Other** |
| CD4+ CD25- Th primary | H3K4me1 | 4.31 | 24.42 | 5.66(2.18) | **0.02934** | **Hematopoietic/Immune** |
| Neurosphere | H3K27ac | 3.15 | 23.07 | 7.32(2.7) | **0.02956** | **CNS** |
| CD4+ CD25- IL17- PMA Ionomycin stim MACS Th sprimary | H3K4me1 | 5.24 | 25.25 | 4.82(1.79) | **0.03071** | **Hematopoietic/Immune** |
| CD25- CD45RA+ naive | H3K27ac | 2.72 | 20.99 | 7.72(3.14) | **0.03307** | **Hematopoietic/Immune** |
| Liver (BI) | H3K9ac | 1.41 | 20.12 | 14.28(6.06) | **0.03325** | **Liver** |
| Fetal heart | H3K4me1 | 4.15 | 19.89 | 4.8(1.92) | **0.04189** | **Cardiovascular** |
| Adipose nuclei | H3K4me1 | 8.79 | 39.34 | 4.48(1.72) | **0.04419** | **Other** |
| Fetal thymus | H3K4me1 | 3.62 | 19.45 | 5.38(2.23) | **0.0448** | **Hematopoietic/Immune** |
| CD3 primary (BI) | H3K4me1 | 2.99 | 17.31 | 5.78(2.47) | **0.04569** | **Hematopoietic/Immune** |
| CD4+ CD25- CD45R0+ memory primary | H3K4me1 | 3.45 | 19.4 | 5.62(2.36) | **0.04729** | **Hematopoietic/Immune** |
| Duodenum Mucosa | H3K4me1 | 4.83 | 25.08 | 5.19(2.12) | **0.04812** | **Gastrointestinal** |
| Duodenum mucosa | H3K27ac | 2.59 | 22.49 | 8.7(3.74) | 0.05003 | Gastrointestinal |
| Th1 | H3K27ac | 3.19 | 16.16 | 5.06(2.12) | 0.05077 | Hematopoietic/Immune |
| CD8 memory primary | H3K4me1 | 3.18 | 17.02 | 5.35(2.28) | 0.05128 | Hematopoietic/Immune |
| CD4 memory primary | H3K4me1 | 4.12 | 20.89 | 5.07(2.11) | 0.05155 | Hematopoietic/Immune |
| CD4+ CD25- CD45RA+ naive primary | H3K4me1 | 3.84 | 19.7 | 5.12(2.18) | 0.05528 | Hematopoietic/Immune |
| Liver (UCSD) | H3K4me3 | 1.19 | 25.76 | 21.65(10.76) | 0.05677 | Liver |
| CD25- IL17- Th stim MACS | H3K27ac | 2.27 | 13.48 | 5.94(2.64) | 0.05677 | Hematopoietic/Immune |
| CD4 naive primary | H3K4me1 | 3.24 | 17.65 | 5.44(2.39) | 0.05899 | Hematopoietic/Immune |
| CD8 naive primary (BI) | H3K4me1 | 3.19 | 19.32 | 6.06(2.71) | 0.06043 | Hematopoietic/Immune |
| Colon smooth muscle | H3K4me1 | 3.52 | 17.14 | 4.87(2.14) | 0.06362 | Gastrointestinal |
| Breast vHMEC | H3K4me1 | 3.43 | 26.62 | 7.76(3.85) | 0.06867 | Other |
| Pancreatic islets | H3K27ac | 2.80 | 13.8 | 4.92(2.15) | 0.0702 | Adrenal/Pancreas |
| Kidney | H3K27ac | 2.51 | 16.67 | 6.63(3.1) | 0.07122 | Kidney |
| CD8 naive primary (UCSF-UBC) | H3K4me1 | 2.48 | 15.21 | 6.13(2.92) | 0.07434 | Hematopoietic/Immune |
| Stomach mucosa | H3K4me1 | 3.36 | 18 | 5.35(2.41) | 0.07733 | Gastrointestinal |
| CD25+ CD127- Treg | H3K27ac | 3.13 | 15.61 | 4.99(2.31) | 0.08376 | Hematopoietic/Immune |
| Colon smooth muscle | H3K27ac | 2.54 | 10.35 | 4.08(1.79) | 0.08403 | Gastrointestinal |
| Adipose nuclei | H3K27ac | 2.65 | 14.3 | 5.4(2.54) | 0.09038 | Other |
| CD3 primary (UW) | H3K4me1 | 3.65 | 16.93 | 4.64(2.2) | 0.0918 | Hematopoietic/Immune |
| Duodenum smooth muscle | H3K27ac | 2.09 | 10.78 | 5.17(2.48) | 0.09434 | Gastrointestinal |
| CD14 primary | H3K4me1 | 4.01 | 18.83 | 4.7(2.26) | 0.09461 | Hematopoietic/Immune |
| Penis foreskin fibroblast primary | H3K4me1 | 7.73 | 27.24 | 3.52(1.58) | 0.10403 | Connective tissue/Bone |
| CD34 primary | H3K4me1 | 3.24 | 18.43 | 5.69(3) | 0.10528 | Hematopoietic/Immune |
| Th0 | H3K27ac | 3.18 | 13.47 | 4.24(2.06) | 0.1074 | Hematopoietic/Immune |
| CD25int CD127+ Tmem | H3K27ac | 3.21 | 16.12 | 5.02(2.6) | 0.12194 | Hematopoietic/Immune |
| Colonic mucosa | H3K27ac | 2.15 | 17.64 | 8.19(4.65) | 0.12414 | Gastrointestinal |
| Treg primary | H3K4me3 | 1.78 | 13.56 | 7.63(4.38) | 0.12443 | Hematopoietic/Immune |
| Rectal mucosa | H3K27ac | 2.20 | 14.54 | 6.61(3.65) | 0.12742 | Gastrointestinal |
| Anterior caudate | H3K4me1 | 3.90 | 17.48 | 4.49(2.31) | 0.12847 | CNS |
| Substantia nigra | H3K27ac | 2.51 | 10.01 | 3.98(1.99) | 0.12878 | CNS |
| Placenta amnion | H3K4me1 | 0.40 | 9.53 | 23.53(15.86) | 0.13269 | Other |
| Fetal heart | H3K9ac | 2.05 | 12.93 | 6.3(3.67) | 0.13981 | Cardiovascular |
| CD14 | H3K27ac | 2.84 | 11.64 | 4.09(2.18) | 0.14309 | Hematopoietic/Immune |
| CD4+ CD25+ CD127- Treg primary | H3K4me1 | 2.86 | 13.58 | 4.75(2.63) | 0.14606 | Hematopoietic/Immune |
| Stomach smooth muscle | H3K27ac | 2.67 | 9.76 | 3.66(1.85) | 0.15877 | Gastrointestinal |
| Gastric | H3K4me1 | 1.36 | 11.6 | 8.53(5.43) | 0.16304 | Gastrointestinal |
| Spleen | H3K4me1 | 4.59 | 19.53 | 4.25(2.44) | 0.16912 | Hematopoietic/Immune |
| Inferior temporal lobe | H3K4me1 | 4.76 | 17.41 | 3.66(1.96) | 0.17069 | CNS |
| Breast myoepithelial | H3K4me1 | 4.76 | 17.17 | 3.61(1.9) | 0.17581 | Other |
| Thymus | H3K4me1 | 0.88 | 7.98 | 9.07(6.12) | 0.17627 | Hematopoietic/Immune |
| Mobilized CD34 | H3K27ac | 3.18 | 15.6 | 4.91(3.07) | 0.17728 | Hematopoietic/Immune |
| Cingulate gyrus | H3K4me1 | 4.10 | 15.68 | 3.82(2.11) | 0.17738 | CNS |
| Adipose nuclei | H3K9ac | 2.86 | 16.71 | 5.84(3.67) | 0.17802 | Other |
| Rectal smooth muscle | H3K27ac | 2.55 | 8.31 | 3.25(1.68) | 0.1843 | Gastrointestinal |
| Th2 | H3K27ac | 3.17 | 11.69 | 3.68(2.09) | 0.18775 | Hematopoietic/Immune |
| Liver (BI) | H3K4me3 | 1.63 | 10.79 | 6.64(4.19) | 0.19548 | Liver |
| Ovary | H3K4me1 | 0.87 | 7.86 | 9.02(6.46) | 0.2033 | Other |
| Breast fibroblast primary | H3K4me1 | 1.03 | 8.86 | 8.59(6.41) | 0.21562 | Connective tissue/Bone |
| Liver (UCSD) | H3K4me1 | 0.15 | 6.51 | 44.66(36.89) | 0.22855 | Liver |
| Fetal large intestine | H3K4me1 | 3.04 | 12.2 | 4.01(2.57) | 0.25321 | Gastrointestinal |
| Hippocampus middle | H3K4me1 | 5.18 | 15.87 | 3.06(1.84) | 0.26057 | CNS |
| CD19 primary (UW) | H3K4me3 | 0.89 | 9.44 | 10.66(8.85) | 0.26653 | Hematopoietic/Immune |
| Rectal mucosa | H3K4me1 | 3.50 | 14.04 | 4.01(2.7) | 0.26709 | Gastrointestinal |
| Mobilized CD34 primary | H3K4me3 | 2.26 | 13.22 | 5.86(4.59) | 0.27401 | Hematopoietic/Immune |
| Left Ventricle | H3K4me1 | 2.66 | 12.2 | 4.58(3.41) | 0.27543 | Cardiovascular |
| CD56 primary | H3K4me3 | 0.84 | 8.46 | 10.05(8.82) | 0.29262 | Hematopoietic/Immune |
| CD25- IL17+ Th17 stim | H3K27ac | 2.72 | 10.65 | 3.92(2.84) | 0.29924 | Hematopoietic/Immune |
| Breast luminal epithelial | H3K4me1 | 0.19 | -3.71 | -19.33(19.91) | 0.30218 | Other |
| Lung | H3K4me1 | 1.75 | 9.5 | 5.42(4.55) | 0.31066 | Cardiovascular |
| CD4+ CD25int CD127+ Tmem primary | H3K4me1 | 1.59 | 7.01 | 4.42(3.53) | 0.32566 | Hematopoietic/Immune |
| CD4+ CD25- IL17- PMA Ionomycin stim MACS Th sprimary | H3K4me3 | 1.72 | 8.6 | 5(4.15) | 0.32716 | Hematopoietic/Immune |
| Fetal small intestine | H3K4me1 | 3.38 | 12.04 | 3.56(2.58) | 0.32945 | Gastrointestinal |
| Fetal placenta | H3K4me1 | 2.53 | 10.2 | 4.04(3.15) | 0.33235 | Other |
| Substantia nigra | H3K4me3 | 1.44 | -7.03 | -4.89(6.26) | 0.33814 | CNS |
| Anterior caudate | H3K27ac | 2.16 | 7.27 | 3.36(2.46) | 0.33957 | CNS |
| Fetal lung | H3K4me1 | 6.99 | 17.37 | 2.48(1.58) | 0.34673 | Cardiovascular |
| Kidney | H3K4me3 | 1.74 | 9.94 | 5.71(4.96) | 0.3476 | Kidney |
| CD14 primary | H3K4me3 | 0.84 | 7.37 | 8.81(8.74) | 0.35469 | Hematopoietic/Immune |
| Mid frontal lobe | H3K4me3 | 1.67 | -7.08 | -4.25(5.74) | 0.35992 | CNS |
| Substantia nigra | H3K4me1 | 3.79 | 10.83 | 2.86(2.06) | 0.36172 | CNS |
| Placenta chorion | H3K4me3 | 0.87 | 8.45 | 9.75(10) | 0.37044 | Other |
| Fetal brain | H3K4me1 | 3.32 | 8.37 | 2.52(1.76) | 0.37987 | CNS |
| Hippocampus middle | H3K27ac | 2.04 | 6.72 | 3.29(2.63) | 0.38092 | CNS |
| Skeletal muscle | H3K4me3 | 2.21 | -5.99 | -2.71(4.41) | 0.39576 | SkeletalMuscle |
| CD15 primary | H3K4me3 | 1.40 | 7.59 | 5.41(5.25) | 0.39588 | Hematopoietic/Immune |
| Penis foreskin fibroblast primary | H3K4me3 | 3.74 | 10.8 | 2.88(2.25) | 0.4005 | Connective tissue/Bone |
| Colon smooth muscle | H3K4me3 | 1.44 | -5.72 | -3.97(5.95) | 0.40078 | Gastrointestinal |
| Penis foreskin melanocyte primary | H3K4me1 | 6.53 | 14.88 | 2.28(1.53) | 0.40613 | Other |
| Psoas muscle | H3K4me1 | 0.39 | 4.31 | 10.96(12.52) | 0.41758 | SkeletalMuscle |
| Skeletal muscle | H3K27ac | 2.37 | -2.4 | -1.01(2.49) | 0.42265 | SkeletalMuscle |
| CD4+ CD25- IL17+ PMA Ionomycin stim Th17 primary | H3K4me3 | 1.26 | 7.24 | 5.74(6.14) | 0.43235 | Hematopoietic/Immune |
| Angular gyrus | H3K4me3 | 1.07 | -5.15 | -4.8(7.58) | 0.44032 | CNS |
| Stomach smooth muscle | H3K4me1 | 2.39 | 8.07 | 3.37(3.08) | 0.44388 | Gastrointestinal |
| Colonic mucosa | H3K9ac | 1.81 | 7.58 | 4.18(4.2) | 0.44779 | Gastrointestinal |
| Gastric | H3K4me3 | 0.59 | 6.12 | 10.35(12.45) | 0.44819 | Gastrointestinal |
| Fetal leg muscle | H3K4me3 | 0.98 | -5.14 | -5.23(8.3) | 0.45377 | SkeletalMuscle |
| Fetal trunk muscle | H3K4me3 | 0.89 | -4.66 | -5.26(8.82) | 0.4729 | SkeletalMuscle |
| Cingulate gyrus | H3K27ac | 2.48 | 6.13 | 2.48(2.09) | 0.47995 | CNS |
| Colonic mucosa | H3K4me1 | 2.17 | 7.42 | 3.42(3.56) | 0.498 | Gastrointestinal |
| Rectal mucosa | H3K9ac | 1.61 | 6.35 | 3.93(4.61) | 0.5227 | Gastrointestinal |
| Pancreatic islets | H3K4me1 | 0.85 | -1.81 | -2.13(4.98) | 0.52605 | Adrenal/Pancreas |
| Fetal large intestine | H3K4me3 | 1.10 | 5.87 | 5.36(6.93) | 0.53112 | Gastrointestinal |
| Duodenum smooth muscle | H3K4me3 | 2.03 | -2.62 | -1.29(3.82) | 0.54515 | Gastrointestinal |
| Rectal smooth muscle | H3K9ac | 0.36 | -2.64 | -7.38(14.21) | 0.5557 | Gastrointestinal |
| Breast vHMEC | H3K4me3 | 0.77 | -3.36 | -4.33(9.11) | 0.55824 | Other |
| Placenta amnion | H3K4me3 | 0.64 | -3.44 | -5.39(11.09) | 0.56391 | Other |
| CD4+ CD25int CD127+ Tmem primary | H3K4me3 | 1.08 | -3.42 | -3.16(7.48) | 0.57167 | Hematopoietic/Immune |
| Pancreas | H3K4me1 | 3.00 | 8.5 | 2.84(3.24) | 0.57173 | Adrenal/Pancreas |
| Fetal placenta | H3K4me3 | 0.83 | 5.38 | 6.52(9.84) | 0.57195 | Other |
| Stomach mucosa | H3K4me3 | 0.59 | 4.92 | 8.32(13.2) | 0.57395 | Gastrointestinal |
| Cingulate gyrus | H3K9ac | 1.92 | 6.16 | 3.21(4.21) | 0.59838 | CNS |
| Mid frontal lobe | H3K4me1 | 0.35 | 2.2 | 6.35(10.57) | 0.6091 | CNS |
| Pancreatic islets | H3K4me3 | 1.14 | 5.49 | 4.83(7.56) | 0.60984 | Adrenal/Pancreas |
| Stomach smooth muscle | H3K9ac | 1.40 | 4.8 | 3.43(4.87) | 0.61562 | Gastrointestinal |
| Rectal mucosa | H3K4me3 | 1.84 | 6.87 | 3.74(5.55) | 0.61881 | Gastrointestinal |
| Rectal smooth muscle | H3K4me3 | 1.30 | -3.05 | -2.34(6.74) | 0.62092 | Gastrointestinal |
| Spleen | H3K4me3 | 0.42 | 3.4 | 8.14(14.78) | 0.6247 | Hematopoietic/Immune |
| Small intestine | H3K4me1 | 0.10 | 1.78 | 17.08(33.7) | 0.6304 | Gastrointestinal |
| Sigmoid colon | H3K4me3 | 0.56 | -2.59 | -4.65(11.98) | 0.63337 | Gastrointestinal |
| Fetal adrenal | H3K4me1 | 4.34 | -1.05 | -0.24(2.64) | 0.63356 | Adrenal/Pancreas |
| Kidney | H3K4me1 | 0.85 | 3.46 | 4.08(6.55) | 0.63618 | Kidney |
| Fetal trunk muscle | H3K4me1 | 4.14 | -0.46 | -0.11(2.44) | 0.64261 | SkeletalMuscle |
| Inferior temporal lobe | H3K4me3 | 1.75 | -2.72 | -1.55(5.53) | 0.64312 | CNS |
| Pancreatic islets | H3K9ac | 0.62 | 4.08 | 6.53(12.28) | 0.64829 | Adrenal/Pancreas |
| Esophagus | H3K4me1 | 0.58 | 3.25 | 5.62(10.3) | 0.65144 | Gastrointestinal |
| Penis foreskin keratinocyte primary | H3K4me3 | 1.92 | -2.49 | -1.3(5.1) | 0.65227 | Other |
| Inferior temporal lobe | H3K9ac | 1.68 | 5.37 | 3.18(4.89) | 0.65379 | CNS |
| CD8 naive primary (UCSF-UBC) | H3K4me3 | 0.62 | 3.48 | 5.63(10.68) | 0.65948 | Hematopoietic/Immune |
| CD3 primary (UW) | H3K4me3 | 1.06 | 4.1 | 3.86(6.61) | 0.6598 | Hematopoietic/Immune |
| Placenta chorion | H3K4me1 | 0.75 | 3.06 | 4.06(7.02) | 0.66254 | Other |
| Sigmoid colon | H3K4me1 | 0.30 | 2.08 | 6.9(14.05) | 0.66935 | Gastrointestinal |
| Rectal smooth muscle | H3K4me1 | 0.93 | 2.8 | 3.02(4.8) | 0.67236 | Gastrointestinal |
| Stomach smooth muscle | H3K4me3 | 2.01 | -2.07 | -1.03(4.88) | 0.67472 | Gastrointestinal |
| Angular gyrus | H3K4me1 | 1.32 | 3.71 | 2.81(4.53) | 0.68707 | CNS |
| Kidney | H3K9ac | 1.08 | 3.72 | 3.46(6.23) | 0.69096 | Kidney |
| Peripheralblood mononuclear primary | H3K9ac | 1.32 | 4.08 | 3.09(5.78) | 0.71409 | Hematopoietic/Immune |
| Pancreatic islets | H3K4me1 | 0.63 | 2.24 | 3.57(7.08) | 0.71457 | Adrenal/Pancreas |
| Skeletal muscle | H3K9ac | 2.76 | 6.14 | 2.22(3.45) | 0.72123 | SkeletalMuscle |
| Small intestine | H3K4me3 | 0.49 | -1.9 | -3.84(13.84) | 0.72142 | Gastrointestinal |
| Fetal small intestine | H3K4me3 | 1.12 | 3.71 | 3.32(6.57) | 0.72499 | Gastrointestinal |
| CD8 primary | H3K4me3 | 1.04 | 3.34 | 3.22(6.53) | 0.73047 | Hematopoietic/Immune |
| Anterior caudate | H3K9ac | 1.46 | 4.14 | 2.83(5.44) | 0.73436 | CNS |
| Aorta | H3K4me3 | 0.85 | 3.44 | 4.04(9.1) | 0.73558 | Cardiovascular |
| Penis foreskin melanocyte primary | H3K4me3 | 1.79 | -0.86 | -0.48(4.43) | 0.7377 | Other |
| CD8 naive primary (UCSF-UBC) | H3K9ac | 0.22 | 1.53 | 6.99(18.07) | 0.73827 | Hematopoietic/Immune |
| Breast fibroblast primary | H3K4me3 | 0.58 | -1.45 | -2.49(10.53) | 0.74063 | Connective tissue/Bone |
| Anterior caudate | H3K4me3 | 1.92 | -1.31 | -0.68(5.11) | 0.74126 | CNS |
| Duodenum smooth muscle | H3K4me1 | 0.60 | -1.31 | -2.19(9.83) | 0.74456 | Gastrointestinal |
| Duodenum Mucosa | H3K9ac | 1.51 | 3.81 | 2.52(4.74) | 0.74666 | Gastrointestinal |
| CD8 memory primary | H3K4me3 | 1.11 | 3.51 | 3.16(6.92) | 0.7519 | Hematopoietic/Immune |
| CD34 primary | H3K4me3 | 1.07 | -1.33 | -1.25(7.25) | 0.75546 | Hematopoietic/Immune |
| Right ventricle | H3K4me1 | 0.08 | 0.73 | 8.98(26.28) | 0.7585 | Cardiovascular |
| Skeletal muscle | H3K4me1 | 6.68 | 9.95 | 1.49(1.62) | 0.75983 | SkeletalMuscle |
| Fetal lung | H3K9ac | 1.30 | -0.96 | -0.74(5.98) | 0.77186 | Cardiovascular |
| Angular gyrus | H3K9ac | 1.10 | -0.92 | -0.84(6.55) | 0.77682 | CNS |
| Psoas muscle | H3K4me3 | 0.99 | -1.35 | -1.36(8.4) | 0.77699 | SkeletalMuscle |
| Fetal heart | H3K4me3 | 0.58 | -0.95 | -1.63(9.39) | 0.77924 | Cardiovascular |
| Right atrium | H3K4me3 | 1.11 | -1.16 | -1.04(7.34) | 0.7805 | Cardiovascular |
| Right ventricle | H3K4me3 | 1.08 | -1.3 | -1.2(7.96) | 0.78144 | Cardiovascular |
| Fetal adrenal | H3K4me3 | 0.98 | -1.26 | -1.28(8.72) | 0.79304 | Adrenal/Pancreas |
| Esophagus | H3K4me3 | 0.87 | -1.22 | -1.4(9.34) | 0.7965 | Gastrointestinal |
| Colon smooth muscle | H3K9ac | 0.57 | -0.73 | -1.29(9.3) | 0.80602 | Gastrointestinal |
| Osteoblast | H3K27ac | 2.86 | 1.45 | 0.51(2.02) | 0.80622 | Connective tissue/Bone |
| Hippocampus middle | H3K4me3 | 1.89 | -0.42 | -0.22(5.06) | 0.8077 | CNS |
| Fetal kidney | H3K9ac | 0.50 | -0.83 | -1.66(11.03) | 0.81007 | Kidney |
| Germinal matrix | H3K4me3 | 1.16 | 3.18 | 2.75(7.44) | 0.81331 | CNS |
| CD4+ CD25- Th primary | H3K4me3 | 1.27 | -0.48 | -0.37(6.26) | 0.82387 | Hematopoietic/Immune |
| Left Ventricle | H3K4me3 | 0.88 | -0.81 | -0.92(8.74) | 0.82539 | Cardiovascular |
| Fetal brain | H3K9ac | 1.27 | -0.26 | -0.2(6.02) | 0.84143 | CNS |
| Stomach mucosa | H3K9ac | 1.31 | 2.69 | 2.05(5.58) | 0.84969 | Gastrointestinal |
| Breast myoepithelial | H3K9ac | 0.46 | -0.73 | -1.58(13.77) | 0.85042 | Other |
| Fetal stomach | H3K4me1 | 3.43 | 5.16 | 1.5(2.72) | 0.85234 | Gastrointestinal |
| Inferior temporal lobe | H3K27ac | 2.60 | 1.5 | 0.58(2.3) | 0.85317 | CNS |
| Mid frontal lobe | H3K9ac | 1.51 | 0.06 | 0.04(5.36) | 0.85676 | CNS |
| CD19 primary (BI) | H3K4me3 | 1.21 | 2.58 | 2.13(6.45) | 0.85962 | Hematopoietic/Immune |
| Fetal thymus | H3K4me3 | 0.93 | 2 | 2.16(6.88) | 0.86542 | Hematopoietic/Immune |
| Pancreatic islets | H3K4me3 | 1.01 | -0.09 | -0.09(6.48) | 0.86628 | Adrenal/Pancreas |
| Fetal stomach | H3K4me3 | 1.02 | 2.28 | 2.24(7.5) | 0.86896 | Gastrointestinal |
| Substantia nigra | H3K9ac | 1.69 | 2.88 | 1.7(4.32) | 0.87023 | CNS |
| Fetal lung | H3K4me3 | 1.07 | -0.07 | -0.06(6.64) | 0.87246 | Cardiovascular |
| Colonic mucosa | H3K4me3 | 1.20 | -0.05 | -0.04(6.67) | 0.87546 | Gastrointestinal |
| Ovary | H3K4me3 | 0.96 | -0.23 | -0.23(8.38) | 0.8825 | Other |
| Lung | H3K4me3 | 0.56 | -0.25 | -0.44(11.52) | 0.89997 | Cardiovascular |
| Adipose nuclei | H3K4me3 | 2.93 | 1.81 | 0.62(3.14) | 0.90168 | Other |
| Angular gyrus | H3K27ac | 2.37 | 3.03 | 1.28(2.42) | 0.90654 | CNS |
| Chondrogenic dif | H3K27ac | 2.65 | 1.94 | 0.73(2.39) | 0.91003 | Connective tissue/Bone |
| Fetal brain | H3K4me3 | 1.29 | 2.22 | 1.72(6.7) | 0.91411 | CNS |
| Fetal brain | H3K4me3 | 0.41 | -0.1 | -0.25(12.71) | 0.92142 | CNS |
| CD4+ CD25+ CD127- Treg primary | H3K4me3 | 1.42 | 2.1 | 1.48(5.7) | 0.93223 | Hematopoietic/Immune |
| CD4 memory primary | H3K4me3 | 1.17 | 0.59 | 0.51(6.2) | 0.93562 | Hematopoietic/Immune |
| Cingulate gyrus | H3K4me3 | 1.77 | 2.52 | 1.42(5.51) | 0.9384 | CNS |
| Penis foreskin keratinocyte primary | H3K9ac | 3.27 | 3.93 | 1.2(2.88) | 0.94351 | Other |
| CD4+ CD25- CD45R0+ memory primary | H3K4me3 | 0.88 | 0.44 | 0.5(7.33) | 0.94532 | Hematopoietic/Immune |
| Peripheralblood mononuclear primary | H3K4me3 | 0.97 | 0.56 | 0.58(8.04) | 0.95778 | Hematopoietic/Immune |
| Peripheralblood mononuclear primary | H3K4me1 | 0.90 | 1.23 | 1.36(7.04) | 0.95836 | Hematopoietic/Immune |
| CD4+ CD25- CD45RA+ naive primary | H3K4me3 | 1.31 | 0.98 | 0.75(5.69) | 0.96405 | Hematopoietic/Immune |
| Duodenum Mucosa | H3K4me3 | 2.02 | 1.69 | 0.84(4.29) | 0.96956 | Gastrointestinal |
| Hippocampus middle | H3K9ac | 1.79 | 2.07 | 1.16(4.45) | 0.972 | CNS |
| Mid frontal lobe | H3K27ac | 1.94 | 1.79 | 0.93(2.86) | 0.97905 | CNS |
| CD8 naive primary (BI) | H3K4me3 | 1.14 | 0.96 | 0.84(7.09) | 0.98139 | Hematopoietic/Immune |
| CD3 primary (BI) | H3K4me3 | 1.42 | 1.24 | 0.88(5.72) | 0.9826 | Hematopoietic/Immune |
| Breast myoepithelial | H3K4me3 | 1.39 | 1.25 | 0.9(6.58) | 0.98788 | Other |
| Fetal leg muscle | H3K4me1 | 4.09 | 4.23 | 1.03(2.33) | 0.98813 | SkeletalMuscle |
| CD4 primary | H3K4me3 | 1.27 | 1.32 | 1.04(6.12) | 0.99412 | Hematopoietic/Immune |
| Pancreas | H3K4me3 | 0.83 | 0.78 | 0.93(9.85) | 0.99455 | Adrenal/Pancreas |
| CD4 naive primary | H3K4me3 | 1.27 | 1.28 | 1.01(5.56) | 0.99891 | Hematopoietic/Immune |
| CNS: central nervous system. When the same cell type in the same histone mark from more than one institution was used, the name of institution is given in parentheses. | | | | | | |
